# Gate-to-grave assessment of plastic from recycling to manufacturing of TENG: a comparison between India and Singapore

**DOI:** 10.1007/s11356-024-33867-w

**Published:** 2024-06-15

**Authors:** Shreya Sharma, Wei Liang Lai, Sunanda Roy, Pradip Kumar Maji, Seeram Ramakrishna, Kheng Lim Goh

**Affiliations:** 1 Newcastle Research & Innovation Institute (NewRIIS), 80 Jurong East Street 21, #05-04, Singapore, 609607 Singapore; 2 Faculty of Science, Agriculture and Engineering, Newcastle University, Newcastle Upon Tyne, NE1 7RU UK; 3 Department of Inorganic Chemistry, University of Oxford, S Parks Rd, Oxford, OX1 3QR UK; 4 School of Engineering, Republic Polytechnic, 9 Woodlands Ave 9, Singapore, 738964 Singapore; 5 Mechanical Engineering, Alliance University, Bangalore, 562106 India; 6 Department of Polymer and Process Engineering, Indian Institute of Technology Roorkee, Saharanpur Campus, Saharanpur, Uttar Pradesh 247001 India; 7 Department of Mechanical Engineering, National University of Singapore, Singapore, 117576 Singapore

**Keywords:** Waste management, Plastic recycling, Life cycle analysis, Environmental assessment, Recycling to manufacturing, Triboelectric nanogenerators

## Abstract

This study assesses the viability of recycled plastic-based triboelectric nanogenerators (TENGs) for sustainable energy harvesting in India and Singapore, concurrently examining plastic waste management. Using material flow analysis and life cycle assessment, the findings revealed that in Singapore, waste-to-energy incineration has a lower environmental impact than landfilling and mechanical recycling, attributed to natural gas usage. In India, recycling offsets impacts from incineration and landfilling, contributing to a lower net environmental impact. Economic performance of a TENG module from PET recyclates showed a 20% carbon footprint reduction when scaling up from lab to industrial “freeze-drying” processes. Key challenges in TENG manufacturing processes are also assessed for future development. This research highlights the potential of recycled plastic-based TENGs in sustainable energy and waste management.

**Supplementary Information:**

The online version contains supplementary material available at 10.1007/s11356-024-33867-w.

## Introduction

Plastics and other petrochemicals will contribute to almost half of the projected oil demand growth and 15% of the global carbon budget by 2050 (Editorial 2022). Currently, plastics stand as one of the four primary materials alongside cement, wood, and steel (Chen et al. 2019). The prevalence of plastics has raised environmental concerns at every stage of their lifecycle, from production to disposal (Chen et al. 2019). The inherent durability in plastic design enable them to persevere in the environment for long periods of time and become widely dispersed (Bergmann et al. 2022). Managing end-of-life (EoL) plastic waste is a global challenge. Approximately half of plastics produced worldwide since 1980 have been improperly disposed on land, increasing the risk of ocean pollution (Ferronato and Torretta 2019; OECD 2022). Mismanaged plastic waste is a primary issue in less developed countries due to less effective waste management systems, with Asia alone contributing to 71% of mismanaged plastics (Ren et al. 2021a). India with a population size similar to China is projected to become the 5th highest contributor of marine plastic pollution by 2025 (MoHUA 2021). In contrast, Singapore, with a population size 243 times smaller than India, ranks among the top 10 countries in plastic waste production per capita while also excelling in safe plastic waste processing (WWF Singapore 2022; World Population Review 2024).

Several life cycle assessment studies have investigated the environmental impact of EoL plastic treatment (Ren et al. 2021b; Tamoor et al. 2022; Venkatachalam et al. 2022). The challenge is to identify the most environmentally promising and economically viable method for plastic waste treatment. Recycling is often regarded as a preferable alternative to incineration and landfilling (Venkatachalam et al. 2022). In a previous study, we discussed how the economic value of plastics decreases with each mechanical recycling cycle (Lai et al. 2022). We also explored the potential of the triboelectric nanogenerator (TENG) as a more sustainable option, allowing for the harvesting of clean, green energy (i.e., electricity) using TENG devices made from recyclates (Lai et al. 2022). TENG harvests mechanical energy from human body and environmental motion, converting it into electrical energy (Lai et al. 2022). TENG is a promising technology for renewable energy generation due to its low manufacturing cost, simple design, high output performance at low frequencies, and material selection flexibility (Lai et al. 2022).

This study aims to conduct a life-cycle analysis (LCA) on common waste plastic EoL treatments in Indian and Singapore. To our knowledge, there are no reports on a comparative analysis of the LCA on the waste plastic EoL treatments in both developed (Singapore) and less developed (India) countries in the literature. The study assessed the sustainability of TENG for both small-scale power electronics and large-scale energy harvesting units through a LCA on TENG fabrication. Given the potential of TENG to reduce plastic waste and contribute to a closed-loop economy, it is crucial to evaluate the environmental impact and carbon footprint of TENG to address potential new environmental challenges.

The study conducted material flow analysis (MFA) of plastics in both countries using national statistics and performance data from two agencies, notably National Environment Agency (NEA) for Singapore, and Central Pollution Control Board (CPCB) for India. The MFA helps identify hotspots in plastic waste management, including plastic stocks (within different processes) and flows (between different processes) within the system, and forecast interactions among various plastic waste streams throughout the value chain (Mehta et al. 2022). We modeled material flows using economic input–output models, which also include economic accounts for plastic waste treatment and TENG manufacturing production and demand. An extended input–output economic model is provided for TENG manufacturing.

In summary, this study extends our previous research by the following: (1) reviewing various plastic waste treatment methods, (2) performing a life-cycle analysis (LCA) from plastic recycling to TENG manufacturing, (3) analyzing material flow and energy consumption in plastic recycling and TENG manufacturing, and (4) assessing environmental and health impacts in these processes to provide recommendations for transitioning to a circular plastic economy.

## Methods and approach

### Overview of life-cycle analysis

To offer an LCA perspective on plastic waste treatment and re-manufacturing (gate-to-grave), we created LCA models by considering the respective countries (Singapore and India) as a “Whole system.”

A combination of MFA-based methodology has been applied to assess the plastic stocks and flows, and environmental impacts are evaluated for the system described by the MFA. Generally, after collecting the total plastic waste in the Singapore (≈ 868,000 tonnes in 2020) and India (≈ 8,600,000 tonnes in 2019), the plastic waste management processes consist of collection, materials recovery facilities (MRFs), mechanical recycling plants, waste-to-energy (WTE) plants, and landfill (Ministry of Housing and Urban Affair 2019; National Environment Agency 2017). Given the growing number of plastics entering the environment each year due to population growth and increased demand for plastic products, we considered various waste treatment technologies to assess their environmental impacts (Sharma et al. 2021).

Subsequently, we conducted an EoL assessment for the TENG device to explore opportunities for circular economy integration after disposal. We assessed for the environmental impact when employing the recycled plastic materials for manufacturing of the TENG device for clean energy production. LCA was performed using primary laboratory data; no further considerations on process improvements are made. A material flow assessment quantified plastic material flow in the LCA model. The results from the data served as the basis for scaling up the process and identifying environmental hotspots. The laboratory process was then compared to conventional production to determine its environmental competitiveness. Where new processes were not competitive, we re-evaluated the environmental feasibility. The lab-scale process was adjusted for industrial applications, incorporating necessary assumptions for building a realistic scenario.

In this study, “MRF” represents materials recovery facilities that sort and categorize waste and recyclables (e.g., glass, metal, paper, and plastics). “Recycling” refers to the mechanical recycling of plastic waste, and “WTE” indicates thermal processing to convert plastic waste into electrical energy or power.

### Goals and scope definition

The aim of this LCA study is to assess the environmental impact of key plastic waste treatments in India and Singapore. To achieve this, we (1) quantified the mass balance of plastics in the existing plastic waste management systems in the two countries, Singapore and India, followed by modeling and comparing the environmental effects of the defined system processes, including mechanical recycling, incineration, and landfilling for plastic waste; (2) pinpointed critical processes and materials responsible for environmental impacts; and (3) guided improvements in plastic waste management by proposing a new emerging technology TENG and performing LCA by modeling the technology at a future, more developed phase.

The functional unit serves as the reference basis for all calculations (i.e., inputs and outputs data) in the LCA model. For nation-wide LCA studies, we considered that the amount of plastic waste output was generated based on a specific geographical region or boundary. Therefore, the functional unit is the gross amount of waste generated in Singapore (≈ 868,000 tonnes in 2020) and India (≈ 8,600,000 tonnes in 2019). However, we have generalized the data (as shown in the life-cycle inventory data) by considering the functional unit of 1 kg of plastic waste or recyclables to serve as a basis reference. The life cycle impact (LCI) data will be used for quantitative evaluation to assess the impact to the environment attributed by each sector or waste management process.

The LCA scope was generalized to cover the geographical boundaries of Singapore and India, respectively, as a “Whole system.” It includes plastic waste management stages such as collection, sorting, mechanical recycling, WTE, and landfilling. Additionally, the scope extends to the use of recycled plastic materials (e.g., PET) in TENG device manufacturing for clean energy production. The findings offer guidance for policymakers and investors seeking to reduce the environmental impact of plastic waste end-of-life (EoL) treatments, which consist of mechanical recycling, co-processing in cement kilns, WTE, and landfills.

### System boundary

**Fig. 1:**
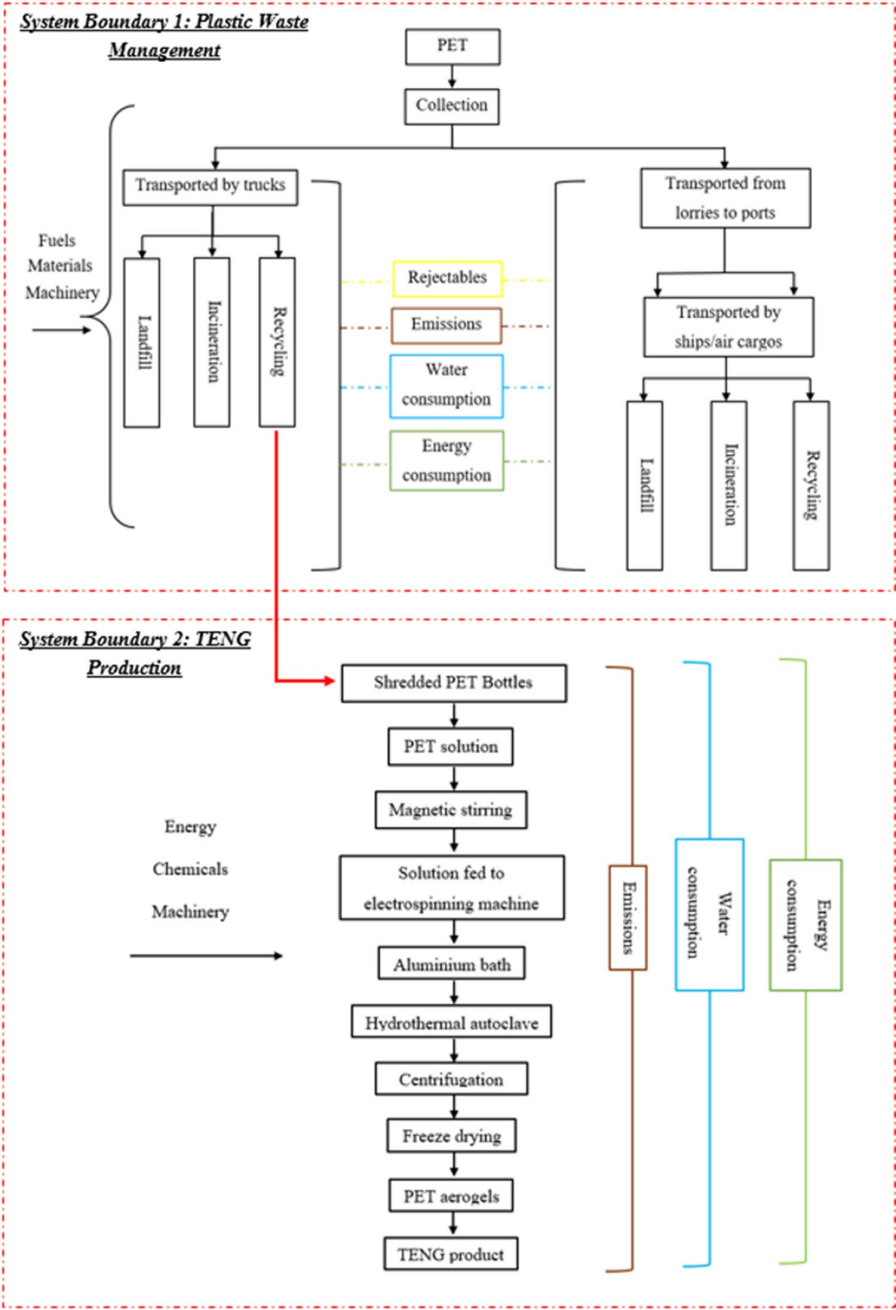
System boundaries for plastic waste management in Singapore and India

Figure 1 shows the system boundary that accounts for the entire plastic waste management process of PET bottles in Singapore and India. The system boundary consists of the vital processes of plastic waste management, EoL treatment, and the potential alternative plastic waste management processes. A “gate to grave” model was adopted to assess the environmental impacts and energy consumptions of the respective plastic treatment processes. The system boundary starts when the consumer/people disposed the PET bottles into the disposal/recycling bin (“from the gate”) and to the final disposal/treatment sites (“to grave”). The production of the materials from cradle was not considered in this study.

The plastic waste management processes included collection and transportation of plastic waste and recyclables from disposal location to the MRF (Transportation i) and MRF to plastic waste/treatment facilities (Transportation ii). The plastic waste/recyclable treatment processes included (a) segregation of polymer types in the MRF, (b) mechanical recycling to process the plastic waste/recyclable (includes grinding, washing, separating, and drying, and treatment processes for the rejectable) to flakes or granules for manufacturing into new products, (c) WTE incineration by thermal processing the plastic waste and using the hot steam to generate electricity for energy recovery, and (d) landfill the plastic waste by disposing to a specific boundary. In addition, India has also co-processed plastic waste in cement kilns by thermal processing to recover materials used for constructions. For further information of each waste management or treatment processes, refer to our previous study (Lai et al. 2022).

### Material flow analysis

The material flow analysis (MFA) approach was used to track the physical flows of the plastic waste or recyclables in India and Singapore. A Sankey diagram was developed using open-source software (STAN 2) to model the flow of the plastic waste and recyclable based on the principle of mass conservation (i.e., input mass is equal to the output mass) (Brunner and Rechberger 2004). The mass of plastics flowing in the MFA systems were quantified in terms of mean value and standard deviation. The standard deviation was considered in the MFA systems to account for plastic leakage or loss during the process and from one process to another.

In this study, the MFA model was built based on the maximum data availability obtained through data collection in both Asian countries for the year 2018. The focus of the MFA closely follows the plastic waste management and treatment processes as shown in the system boundary (see Fig. 1).

The MFA quantified plastic material movement across plastic waste management processes, spanning from raw plastic production to waste treatment. The MFA considered total mixed plastics (including PET plastics) to assess various aspects of the circular economy, with an emphasis on reducing, reusing, and recycling PET plastics.

Table 1 shows the material flow inventory which constitute the data that has been collected from a wide range of sources (i.e., government report, web, journal articles) for different processes. Of note, the environmental impacts of the system described by the MFA were assessed using LCA.

**Table 1:** Material flow inventory

Processes	Country	Component	Data	Source
Generation	Singapore	Total plastic generation	1.76 billion plastic items	(Teo 2018)
		Total PET generation	467 million PET bottles	(Singapore Environment Council 2021)
	India	Total plastic generation	15.12 million tonnes	(Kapur-Bakshi et al. 2021)
		Total PET generation	2.25 million tonnes	-
Import	Singapore	Imported virgin plastic good	Undisclosed mass (291 billion plastic items)	-
	India	Imported virgin plastic good	-	-
Waste	Singapore	Plastic waste generated	-	-
	India	Plastic waste generated	8.76 million tonnes	-
Export	Singapore	Exported virgin plastic good	-	-
		Exported plastic waste	42,000 tonnes	(CNA 2020)
	India	Exported virgin plastic good	-	(Plastindia Foundation 2019)
		Exported plastic waste	-	-
Collection and transport	Singapore	Collected plastic waste	868,000 tonnes	(National Environment Agency 2021a)
	India	Collected plastic waste	5.26 million metric tonnes	-
Treatment	Singapore	Recycling	36,000 tonnes	(National Environment Agency 2021b)
		Incineration	832,000 tonnes	(National Environment Agency 2021b)
		Landfill	86,800 tonnes	(National Environment Agency 2021b)
	India	Recycling	3.68 million tonnes	(Centre for Science and Environment 2020)
		Incineration	0.126 million tonnes	(Centre for Science and Environment 2020)
		Co-processing in cement kilns	0.14 million tonnes	(Centre for Science and Environment 2020)
		Landfill	Data not available	Data not available

### Life cycle inventory

For the LCA, we adopted two approaches: (1) assessment of the environmental impacts of the EoL treatments commonly practiced in both the countries, Singapore and India; (2) prospective life cycle analysis for the proposed emerging technology, TENG, where the assessment has been done by modeling the technology at a future, more developed phase.

#### Life cycle inventory for plastic waste management in Singapore and India

The inventory data was based on data collection of the plastic waste management processes, EoL treatment, and transport distances followed by documentation of the data collected covering the inputs-outputs. For Singapore, the input–output data for the respective mechanical recycling (Table S1) and incineration (Table S2) was obtained from Khoo et al. and National Environmental Agency (NEA) (Khoo 2019; National Environment Agency 2022b). For India, the input–output data for the mechanical recycling (Table S1) was obtained from Central Pollution Control Board (CPCB) and national reports by Aryan and co-workers (Aryan et al. 2019; Central Pollution Control Board 2018; Central Public Health and Environmental Engineering Organisation, Ministry of Urban Developments 2017). For both India and Singapore, the input–output data for landfill (Table S3) was not readily available and henceforth was estimated based on the data from previously published studies (Obersteiner et al. 2007; Tan and Khoo 2006). In India, a proportion of the collected plastic waste (8%) was co-processed in cement-kilns; we have considered this using the data provided by Kosajan and co-workers (Table S4) (Kosajan et al. 2020).

Transport distances included the distance traveled by local transports from waste collection/disposal points to MRF/traders and to the different plastic waste treatment sites as well as distance traveled in exporting of plastic waste. Singapore does not import plastic waste, and since India has banned the import of plastic waste since August 2019, the input–output data for importing of plastic waste was not considered. The plastic scraps have been exported to neighboring countries for recycling in Singapore whereas India was able to house all the recycling processes and facilities in the country (Kerdlap et al. 2021; Ministry of Housing and Urban Affair 2019). Therefore, the exported plastic waste was only considered for Singapore. The furthest of the sea distance from Singapore to Malaysia, Vietnam, and Indonesia was taken as the average export distance. We estimated local transportation distances from published literature as well as through mapping tools such as Google maps (Aryan et al. 2019; Khoo 2019). Euro VI (Bharat VI in India) emission standards were considered for calculating the output data for both Singapore (Table S5) and India (Table S6) (IICT Policy Update 2016; Williams and Minjares 2016). For Singapore, the data of air emissions was also reported for sea transportation methods for waste disposal to the offshore landfill (known as Palau Semakau or Semakau island).

#### Life cycle inventory for upcycling of plastic to TENG manufacturing

For upcycling to manufacture TENG, we considered two levels of manufacturing, namely laboratory- and industrial-scale. Tables S7 and S8 show the energy inventory and material inventory, respectively, as defined by the system boundary. The TENG manufacturing processes contributed to a direct emission from the cleaning solvents and wastewater (in mass). Typically, electricity was utilized to operate the equipment for manufacturing of TENG. Therefore, the total energy consumption through the entire manufacturing process of TENG was calculated by summing the equipment-rated power multiply by the operating time of each instrument. Of note, we estimated the process emissions based on the coal thermal plants for India and natural gas plants for Singapore.

From our estimation from a previous study (Roy et al. 2021), we found that approximately 400 tonnes of PET waste was needed to produce 1 tonne of aerogel. For producing the PET aerogel in a laboratory-scale setup, the TENG manufacturing processes and equipment used were defined in our previous study (Roy et al. 2021). For industrial-scale setup, the energy inventory of manufacturing processes was based on findings of data obtained from research articles, and expert reviews (see Table S7). Case studies and literature reviews resulted in identification of potential key challenges, to which the study aims to provide solutions obtained either through studying established large-scale frameworks or by identifying the best practices in industry to build a realistic scenario.

The data in this section were primarily collected from various sources, such as reports and literature. In cases where data were unavailable, assumptions were made and are outlined in Table S9.

### Impact assessment

In this study, open-source software, OpenLCA, was used to develop and simulate the LCA model, following the ISO 14040 and 14044 standards (Laurent et al. 2014; Loh et al. 2020). The LCA model evaluated the environmental impact of plastic waste management and treatment processes, focusing on factors like energy consumption, energy generation, and gas emissions.

A total of 18 different environmental impact categories were considered. The cumulative energy demand (CED) approach was used to assess the usage of the direct and indirect energy consumption and generation. The ReCiPe 2016 and Impact 2002 + methods were used to assess the seventeen other relevant environmental impact categories. With regard to ReCiPe 2016 method, both mid-point and end-point approach were employed to assess the environmental impact to apprehend the full picture of environmental effects.

CED uses impact indicators to evaluate the energy consumption throughout the life cycle of a service or a product and therefore it can be established with environmental impacts with regard to energy resource depletion (Cascone et al. 2020). Both non-renewable and renewable sources of energy were accounted for calculating the CED. ReCiPe method was commonly used in LCA studies to consider any possible differences in the results and fostering robustness of data and impact assessment results (Jeswani et al. 2021). The ReCiPe method consists of two approaches, namely at midpoint level and at endpoint level for deriving the characterization factors. The characterization factors at the midpoint level were located at a point along the cause-impact pathway, typically at the point after which the environmental mechanism is identical for each environmental flow assigned to that impact category (Huijbregts et al. 2017). The endpoint level simplifies the interpretations of midpoint characterization results based on three aggregation levels, which includes damage to human health, damage to resource availability, and damage to ecosystems.

For comparative assessment of eco-toxicity and human toxicity, Impact 2002 + method has been utilized. Both ReCiPe and Impact 2002 + methods provide a unified approach to calculate characterization factors for both midpoint and endpoint impacts. In addition, fossil CO_2_ eq., main air pollutants and PM, fossil fuel depletion, and smog impacts were derived with the respective Greenhouse Gas Protocol, Ecological scarcity 2013, and TRACI 2.1 to provide a broader perspective in LCA study.

### Sensitivity analysis

Sensitivity analysis (ISO 14049) was performed to evaluate result reliability, as errors in the LCA and MFA may impact and potentially alter the results. A similar qualitative data assessment approach used by Laner and co-workers were employed in this study to quantify for the coefficients of variation (CV, standard deviation divided by mean value) (Laner et al. 2016). The qualitative data assessment approach uses a pedigree matrix to evaluate (by scoring from 1, good quality, to 4, poor quality) the five different data quality indicators (DQI), namely reliability, completeness, temporal, geographical, and technological representativeness (Laner et al. 2016; Weidema and Wesnæs 1996). Also, different sensitivity levels were defined for expressing the sensitivity of the studied quantity in relation to the deviations in a specific indicator (Van Eygen et al. 2017). Generally, the scoring was given to the DQI based on how well the data generation was documented and the expert judgement of the scorer (Van Eygen et al. 2017).

In this study, we assessed the effect of the results when all DQI were scored 1 for the good quality and compared to the results when all DQI were scored 4 for the poor quality (based on high sensitivity level). Herein, all data were assumed to be normally distributed, and the CV were obtained from an exponential function by Laner et al. (2016; see Table 2). The total CV was used to assess for the error in the LCA model, which is expressed as:

**Table 2:** Coefficients of variation (%) for the data quality indicators, obtained from Laner et al. (2016)

Data quality indicator	Sensitivity level	Score: 1	Score: 2	Score: 3	Score: 4
Reliability	-	2.3	6.8	20.6	62.3
Completeness/temporal/geographical/technological representativeness	High	0	4.5	13.7	41.3
	Medium	0	2.3	6.8	20.6
	Low	0	1.1	3.4	10.3

1 $${CV}_{total}= \sqrt{{{CV}_{reliability}}^{2}+{{CV}_{completeness}}^{2}+{{CV}_{temporal}}^{2}+{{CV}_{geographical}}^{2}+{{CV}_{technological}}^{2}}$$

## Results and discussion

### Overview

In this section, we present the findings from (1) material flow analysis (MFA); (2) life-cycle assessment (LCA) covering (i) plastic waste management processes, and (ii) upcycling of plastic for TENG manufacturing; and (3) techno-economic analysis of PET plastic scraps in two Asian countries, Singapore and India. In MFA, we quantified the plastic material flow within the defined system boundary (Section 2.3). In LCA, we assessed the environmental impact of plastic materials undergoing standard waste and recycling processes. We also evaluated the environmental impact of plastic treatment processes for upcycling to TENG manufacturing, both at the laboratory scale and industrial level. In the techno-economic analysis, we assessed the economic value of various plastic treatment processes and TENG manufacturing. Notably, we conducted comparative analyses between Singapore, a developed high-GDP per capita country, and India, a developing nation with a lower GDP per capita.

### Material flow analysis

**Fig. 2:**
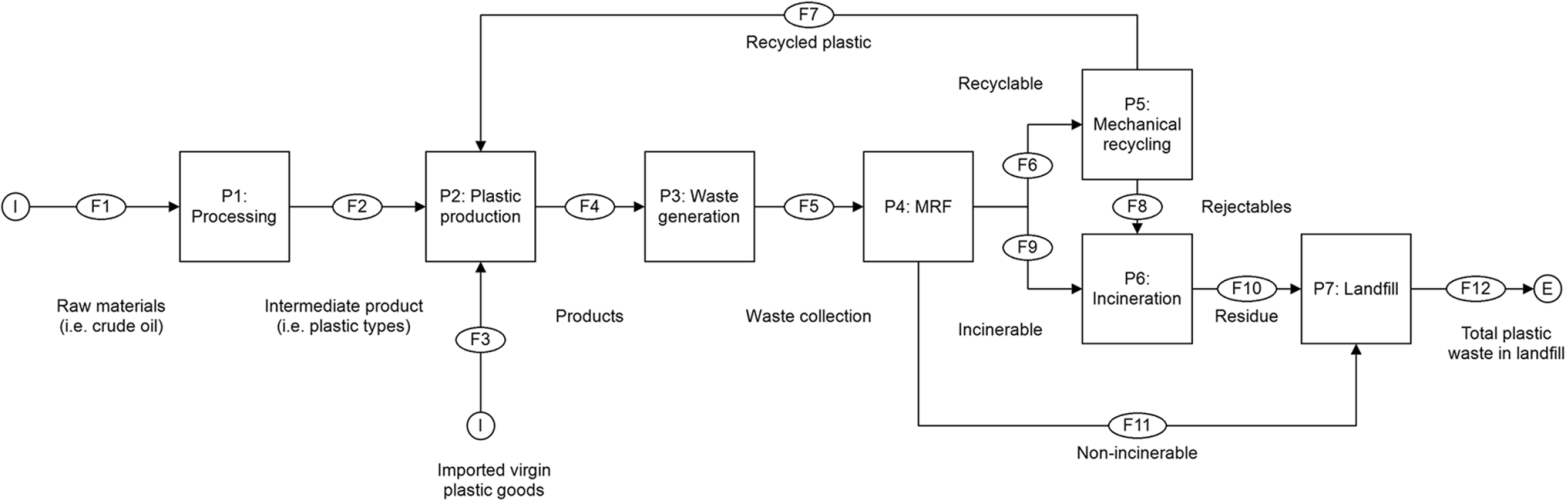
Diagram for material flow analysis of plastics in Singapore

**Fig. 3:**
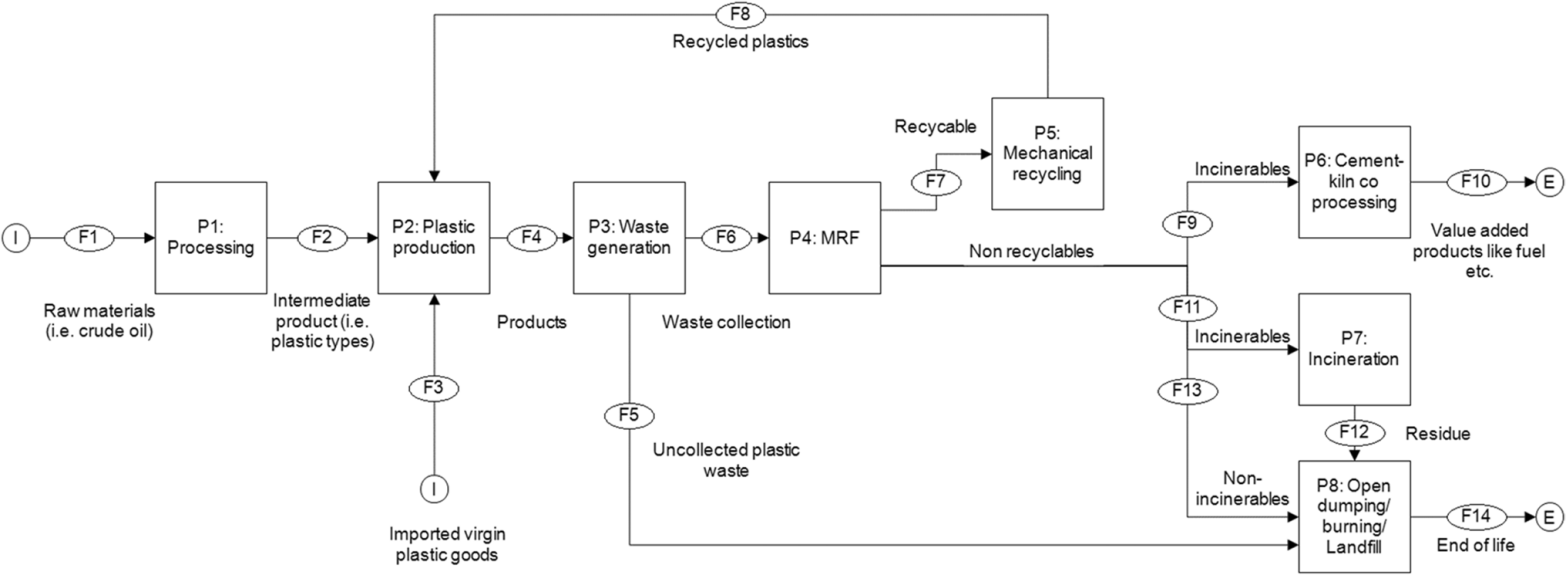
Diagram for material flow analysis of plastics in India

Figures 2 and 3 present the MFA diagrams to illustrate the system boundary of Singapore and India, respectively. Tables 3 and 4 present the estimated value of plastic mass flowing at different process stages in Singapore and India, respectively. Three indicators were employed to describe the extent of plastic material recirculation within the economy: the collection rate (amount collected divided by total waste amount), the sorting rate (amount sorted and sent for mechanical recycling divided by total waste amount), and the recycling rate (amount of re-granulate produced at the mechanical recycling plant divided by total waste amount).

**Table 3:** Mass of plastic flowing at different process stages in Singapore. The mass of plastic flowing into and through the processes were simulated using the STAN 2 software

Material flow	Mass (tons/annum)	Processes	Mass (tons/annum)
F1: Raw materials (i.e., crude oil)	Undisclosed	P1: Processing (crude oil to plastic materials)	Undisclosed
F2: Intermediate product (i.e., plastic type)	Undisclosed (17.76 billion pieces)	P2: Plastic production (Plastic materials to goods/products)	Undisclosed
F3: Imported virgin plastic goods/products	+ 6000	P3: waste generation (plastic goods/products to waste)	0
F4: plastic goods/products	Undisclosed	P4: MRF (waste to different waste treatment)	− 6000
F5: plastic waste collection	868,000	P5: Mechanical recycling (waste to recycled plastics)	0
F6: recyclables	42,000	P6: Incineration (waste to residue)	0
F7: recycled plastics	36,000	P7: landfill (residue to landfill)	− 3000
F8: rejectables from mechanical recycling	6000	• Undisclosed represents mass data not available or found • Values with positive sign ( +) represents the estimated mass that could be contributed to the material flow • Values with negative sign ( −) represents the estimated mass required to be contributed to a material flow to balance the process • The zero value of mass represents that the process system is balanced	
F9: Incinerable plastic waste	832,000		
F10: residue from incinerated plastic waste	83,800 (mass reduction after incineration)		
F11: non-incinerable plastic waste	+ 3000		
F12: total plastic waste in landfill	86,800

**Table 4:** Mass of plastic flowing at different process stages in India. The mass of plastic flowing into and through the processes were simulated using the STAN 2 software

Material flow	Mass (tons/annum)	Processes	Mass (tons/annum)
F1: raw materials (i.e., crude oil)	Undisclosed	P1: processing (crude oil to plastic materials)	Undisclosed
F2: intermediate product (i.e., plastic type)	15,120,000	P2: plastic production (plastic materials to goods/products)	− 6,350,400
F3: imported virgin plastic goods/products	+ 6,350,400	P3: waste generation (plastic goods/products to waste)	0
F4: plastic goods/products	8,769,600	P4: MRF (waste to different waste treatment)	0
F5: uncollected plastic waste	3,507,840	P5: mechanical recycling (waste to recycled plastics)	0
F6: waste collection	5,261,760	P6: cement-kiln co processing	0
F7: recyclable	3683,232	P7: incineration	− 126,282.24
F8: recycled plastics	3683,232	P8: open dumping/burning/landfill	− 4,818,018.24
F9: incinerable plastic waste to cement-kiln co processing	142,067.52	• Undisclosed represents mass data not available or found • Values with positive sign ( +) represents the estimated mass that could be contributed to the material flow • Values with negative sign ( −) represents the estimated mass required to be contributed to a material flow to balance the process • The zero value of mass represents that the process system is balanced	
F10: plastic waste to value added products like fuel etc	142,067.52		
F11: incinerable plastic waste	+ 126,282.24		
F12: residue from incineration plastic waste	12,628.2 (mass reduction after incineration)		
F13: non-incinerable plastic waste	1,310, 178.24		
F14: end of life	+ 4,818,018.24		

For Singapore, the dominant stream comprised of the plastic production with 17.76 billion pieces (F2) of mixed plastic items (i.e., PET, HDPE, LDPE, PVC, PS, and other thermosets and thermoplastics) produced in 2018. Through MFA simulations, it was predicted that the MRF process has a balance stock of 6000 tons which may have contributed by the imported virgin plastic goods (F3). No information on the mass of raw materials (F1) used in processing for making plastics (P1) and the mass of intermediate plastic products (F2) are available. We deduced that the magnitude of raw materials (F1) were likely to be larger than intermediate products or goods (F2) after production (P2), since the plastic materials will be compounded and molded into different types of products or goods. The plastic products (F4) were retailed and sold to the consumers. Upon EoL, the consumer disposed the products into the waste bin or disposal point (P3) and resulted to a total mass of 868 thousand tons of plastic waste being collected (F5) and sent to MRF (P4) for sorting to different waste management sites. Singapore has exported (F6) about 42 thousand tons of the plastic recyclates to its neighboring countries for mechanical recycling (P5), and about 36,000 tons of recycled plastics (F7) were implemented for production of plastic products and goods (P2). Here, the MFA simulation has predicted about 6000 tons of plastic rejectable (F8) that were rejected during the mechanical recycling process (P5). For non-recyclable, about 832,000 tons of incinerable plastic (F9) were sent to the WTE plant and were incinerated to reduce the volume of plastic waste and generate electricity for the countries. Several articles have reported that the incineration process (P6) has reduced the volume of incinerable plastics by 90%, generating about 83.8 thousand tons of residues (F10). Subsequently, the residues and non-incinerable plastic (F11) were sent to offshore Semakau landfill (P7) and were dumped into the designated rock bund that encloses part of the sea. The mass of the non-incinerable plastic has been predicted to be 3000 tons. The total mass of waste (residue and non-incinerable) has been estimated to be 86.8 thousand tons.

Generally, the material flow through the different processes between P1 raw materials processing and P4 MRF in India were similar to Singapore and the differences are the distribution of plastic waste and recyclables to the different treatment processes (from P5 mechanical recycling to P8 landfill). In India, the plastic production of mixed plastic items was about 15.12 million tons in 2018. The MFA simulations have predicted a balance stock of 6.35 million tons that may have been contributed by the imported virgin plastic goods (F3). No information on the mass of raw materials (F1) used for making plastics (P1) were found and we deduced that the magnitude of raw materials (F1) was likely to be larger than intermediate products or goods (F2) after production (P2). The plastic products (F4) were retailed and sold to the consumers. Upon EoL, the consumer disposed the products into the waste bin or disposal point (P3) generating a total plastic waste mass of about 8.77 million tons. However, only 5.26 million tons of plastic waste was collected (F6) and sent to different waste treatment sites. We predicted that the remaining 3.51 million tons of uncollected waste (F5) were disposed illegally by dumping/burnt openly (P8). India has a recycling rate of 70% which is about 3.68 million tons (F7) of plastic recyclables being sent for mechanical recycling (P5) to generate recycled plastic (F8). Of note, the amount of recycled plastic (F8) is assumed to be the same as the plastic recyclables (F7) which gives 100% of plastic recycling efficiency. For non-recyclable plastic, about 1.58 million tons of plastic were distributed to different waste treatment processes, namely P6 cement kiln-coprocessing (F9 142 thousand tons), P7 incineration (F11 126 thousand tons), and P8 landfill (F13 1.31 million tons). CPCB have reported that the incineration process (P7) has reduced the volume of incinerable plastics by 90%, generating about 12.63 thousand tons of residues (F12). Subsequently, the residues, non-incinerable plastic (F13), and uncollected plastic (F5) were openly dumped/burned or sent to landfill (P8) which resulted in a total mass of about 4.82 million tons of plastic waste (F14). India favors cement kiln co-processing (P6) for plastic waste management due to its capacity to generate minimal residue after treatment, recover energy, and produce alternative fuel/material products (M. of E. F. and C. C. G. of I. Central Pollution Control Board, n.d.).

### Environmental assessment of plastic waste management processes in Singapore and India

#### Environmental impact assessment: Singapore

Generally, the plastic waste and recyclable management processes in Singapore consist of (1) used bottles to traders/MRF (indicated as Collection i), (2) bundled PET bottles to PWM sites (indicated as Collection ii), (3) mechanical recycling, (4) incineration, and (5) landfill.

**Fig. 4:**
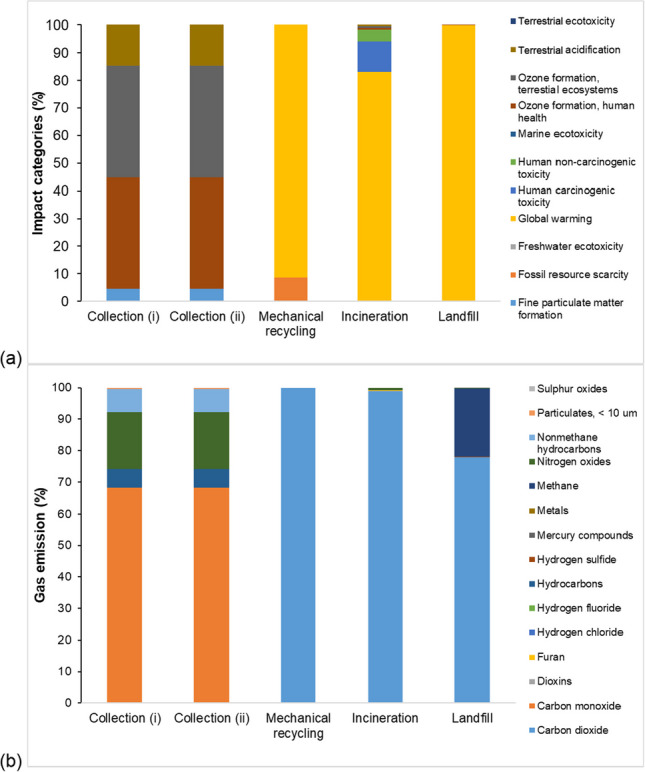
Bar charts show the (**a**) types of environmental impact and (**b**) types of gas emitted by the respective plastic waste and recyclable management processes in Singapore. The results were derived using ReCiPe midpoint (H) method. N.B. Different colors of bar chart represent the different categories of environmental impact and gas types emitted

Figure 4(a) shows the predictions of contributions to the ozone formation in the terrestrial ecosystem (~ 40%) and human health (~ 40%), terrestrial acidification (~ 15%), and fine particular matter formation (~ 5%) by the collection processes i and ii. The contribution from the collection process (i) has negligible environmental effects, as indicated by the negative values (see Supplementary Information, Tables S10 and S11). The mechanical recycling process primarily contributes to global warming (about 91%) and fossil resource scarcity (about 9%). The incineration process is a major contributor to global warming (about 83%), human carcinogenic toxicity (about 11%), and human non-carcinogenic toxicity (about 4%). Additionally, the incineration process has some impact on other environmental factors, although the contributions to terrestrial ecotoxicity and acidification, ozone formation, marine ecotoxicity, freshwater ecotoxicity, fossil resource scarcity, and fine particulate matter formation are negligible. The landfill process is the main contributor to global warming (approximately 99%). Furthermore, the landfill process has some impact on other environmental factors, but the contributions to ozone formation and terrestrial acidification are negligible.

Figure 4(b) indicates that collection processes i and ii release significant carbon monoxide (about 68%), followed by nitrogen oxide (about 18%), NMHC (about 7%), hydrocarbons (about 6%), and particulates (about 0.5). However, the gas emissions from collection process (i) have a negligible environmental impact as the emitted gas amounts are negative (see Supplementary Information, Table S12). The mechanical recycling process primarily emits carbon dioxide (100%) compared to other processes. The incineration process mainly releases carbon dioxide (about 98%), while emissions of other gases (dioxins, furan, hydrogen chloride, mercury compounds, nitrogen oxides, and particulates) are negligible. The landfill process mainly emits carbon dioxide (about 78%) and methane (about 21%), while other emitted gases (carbon monoxide, hydrogen chloride, hydrogen fluoride, hydrogen sulfide metals, nitrogen oxides, and sulfur oxides) have negligible impacts.

**Fig. 5:**
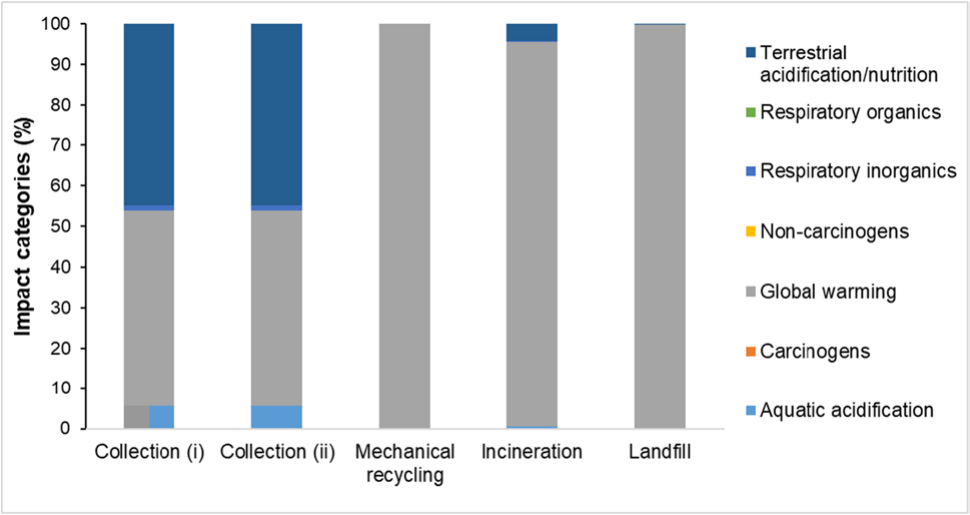
Bar charts of different environmental impact contributed by the respective plastic waste and recyclable management processes in Singapore, derived using Impact 2002 + method. The result presents the impact categories that were not considered by the ReCiPe midpoint (H). N.B. Different colors of bar chart represent the different categories of environmental impact

Figure 5 shows the bar chart of different types of gas emitted to the environment by the plastic waste and recyclable management processes in Singapore. The Impact 2002 + analysis method was used to assess the types of environmental impact that were not considered by the ReCiPe midpoint (H) method (see Supplementary Information, Table S13). The findings show that collection processes (i) and (ii) have primarily contributed to ozone formation in global warming (about 48%), terrestrial acidification/nutrition (about 45%), aquatic acidification (about 6%), and respiratory inorganics (about 1%). However, the contribution from collection process (i) has a negligible environmental impact, as the contributions derived from the analysis are negative (Croes and Vermeulen 2021; Poulopoulos and Inglezakis 2016). The mechanical recycling process primarily contributes to global warming (about 100%). The incineration process is a major contributor to global warming (about 95%), followed by terrestrial acidification/nutrition (about 4%), while other environmental impact contributions (such as aquatic acidification, carcinogens, non-carcinogens, and respiratory inorganics) are negligible.

#### Environmental impact assessment: India

**Fig. 6:**
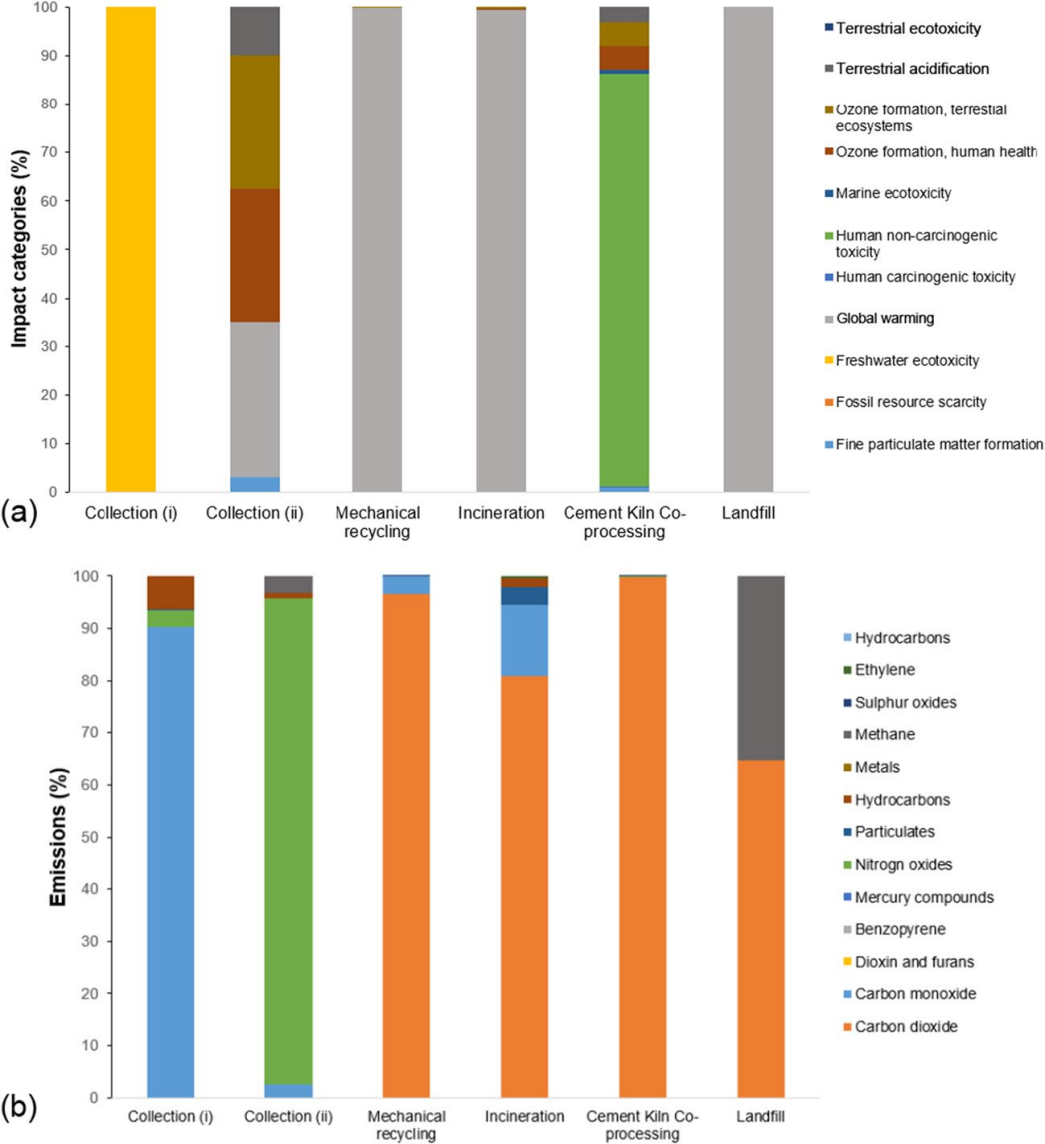
Bar charts show the (**a**) types of environmental impact and (**b**) types of gas emitted by the respective plastic waste and recyclable management processes in India. The results were derived using ReCiPe midpoint (H) method. N.B. Different colors of bar chart represent the different categories of environmental impact and gas types emitted

Figure 6(a) indicates that the contribution of collection process (i) to ozone formation, terrestrial ecosystem, human health, terrestrial acidification, and fine particulate matter formation is almost negligible. Collection process (ii) contributed about 27%, 27%, 9%, and 3% to these categories, respectively. Mechanical recycling, incineration, and landfilling have primarily contributed to global warming (about 100%) and have negligible contributions to other impact categories, including fine particulate matter formation, ozone formation, terrestrial ecosystem, human health, and terrestrial acidification. Co-processing plastic waste in cement kilns resulted in about 85% contribution to human non-carcinogenic toxicity, about 4% contribution to ozone formation, human health, ozone formation, terrestrial ecosystems, and about 3% contribution to terrestrial acidification. For more details and the dataset derived from ReCiPe midpoint and endpoint (H), see Supplementary Information, Tables S14 and S15.

Figure 6(b) reveals that collection process (i) has emitted a significant amount of carbon monoxide (~ 90%), followed by hydrocarbons (~ 6%), and nitrogen oxides (~ 3%). The mechanical recycling process has emitted a substantial amount of carbon dioxide (~ 100%) in comparison to other processes. Collection process (ii) mainly emitted nitrogen oxides (~ 93%), followed by methane (~ 3%), and carbon monoxide (~ 2%). The incineration process displayed large emissions of carbon dioxide (~ 81%), followed by carbon monoxide (~ 13%), particulate formation (~ 3%), and the emission of hydrocarbons (~ 2%). The processing of plastic waste in cement kilns emitted a substantial amount of carbon dioxide (~ 99%). The landfill process primarily emitted carbon dioxide (~ 65%), followed by methane (~ 35%), while the emissions of other gases (carbon monoxide, hydrogen chloride, hydrogen fluoride, hydrogen sulfide, metals, nitrogen oxides, and sulfur oxides) were negligible. For more details and the dataset derived from ReCiPe midpoint (H), see Supplementary Information, Table S16.

**Fig. 7:**
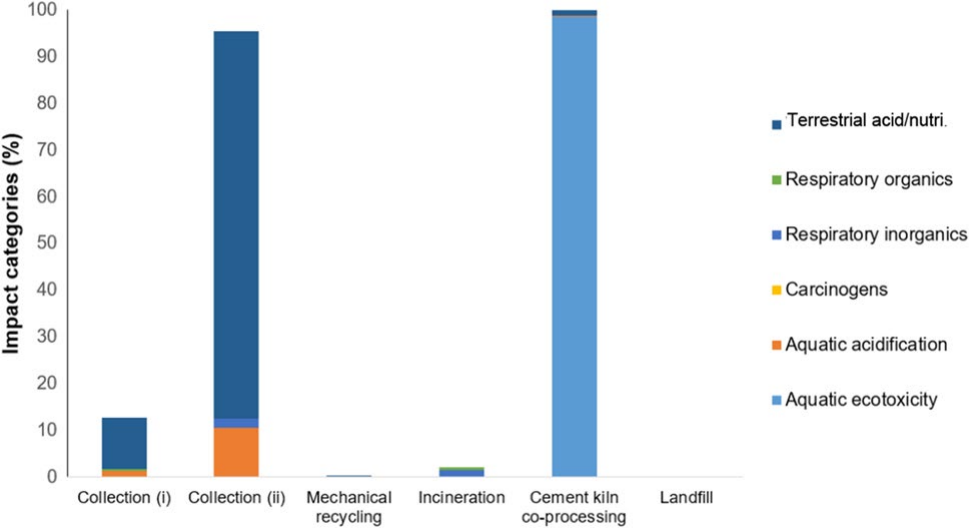
Bar charts of different environmental impact contributed by the respective plastic waste and recyclable management processes in India, derived using Impact 2002 + method. The result presents those impact categories that were not considered by the ReCiPe midpoint (H). N.B. Different colors of bar chart represent the different categories of environmental impact

Figure 7 displays a bar chart illustrating various types of gases emitted into the environment by plastic waste and recyclable management processes in India. The Impact 2002 + analysis method was utilized to evaluate environmental impacts that were not considered by the ReCiPe midpoint (H) method. The results indicate that collection process (i) significantly contributed to global warming (~ 87%), terrestrial acidification/nutrition (~ 11%), and aquatic acidification (~ 1%). In contrast, collection process (ii) mainly contributed to terrestrial acidification/nutrition (~ 82%), aquatic acidification (~ 10%), and global warming (~ 4%). The mechanical recycling process, incineration, and landfilling of plastic waste primarily contributed to global warming. Co-processing of plastic in cement kilns resulted in ~ 98% contributions to aquatic ecotoxicity. For more details and the dataset derived from Impact 2002 + , see Supplementary Information, Table S17.

#### Comparative analysis of environmental assessment between Singapore and India

With regard to gas emissions associated with the collection and transportation of plastic waste, Singapore exhibits a higher environmental impact due to gas emissions from the use of vehicles for transporting used bottles to MRF. In contrast, India heavily relies on the informal sector, which uses man-powered vehicles like pushcarts, tricycles, and electronic rickshaws, resulting in negligible gas emissions. However, the environmental impact related to the transportation of bundled PET bottles to waste management sites is higher in India because of the extensive use of petroleum-powered vehicles. Singapore employs electric vehicles, which are more environmentally friendly as they use little to no fossil fuels, such as petrol or diesel (Castelvecchi 2021; Toh Ting Wei 2021). As a result, India has emitted a substantial amount of greenhouse gas emissions, namely carbon dioxide and carbon monoxide.

Overall, in terms of the environmental impact resulting from the plastic waste management strategies and processes employed in both countries, India exhibits a lower net environmental impact. This is primarily attributed to the recycling processes, which help offset the environmental impact associated with other plastic waste treatment methods, such as incineration or landfilling. When producing recycled plastic granules through mechanical recycling, gas emissions linked to virgin plastic production can be avoided, thanks to the energy- and water-intensive nature of the process. The use of crude oil in virgin plastic production leads to the direct depletion of fossil fuels, an energy source, while also consuming water through high-pressure pumping methods. Additionally, the credit from incineration and co-processing in cement kilns does not significantly contribute to the overall impact due to their limited use in large-scale operations.

In Singapore, the “incineration” process significantly contributes less to the overall environmental impact compared to other processes like landfilling and mechanical recycling. This is due to the primary use of natural gas as the energy source for operating WTE plants that incinerate plastic waste. Moreover, the WTE plant can recover energy from the incineration process, which replaces the electricity (i.e., natural gas) used in the WTE plants. The environmental impact resulting from mechanical recycling is notably higher in Singapore. This is because the country’s in-house mechanical recycling facilities are not well developed, and as a result, the stages involved in mechanical recycling (e.g., sorting, shredding, and cleaning) consume a significant amount of energy, leading to greenhouse gas emissions. Unfortunately, the reliability of LCA and the breakdown of contributions from various plastic waste management technologies could be questionable in the case of India. This is primarily due to the widespread mismanagement of plastic waste through open burning and open dumping, resulting in the release of greenhouse gases and other toxic pollutants into the atmosphere, which often goes unreported.

### Upcycling of plastic to TENG manufacturing

#### Upscaling of TENG manufacturing

To industrialize mass TENG device production using upcycled plastic waste, this requires substantial equipment changes for the transition from the lab to industry. In this study, we gathered information on industrial-scale TENG manufacturing from relevant literature, including various processes and equipment used. We made assumptions, considered pilot-scale models, and explored potential process improvements. Then, we conducted a comparative study to evaluate power consumption and gas emissions across different manufacturing processes. This analysis covered both existing lab equipment and proposed industrial-scale designs (Tables S7 and S8).

Here, we present the types of equipment that have been selected for the different manufacturing processes for the industrial-scale setup. For “stirring” process, we selected the IKA Standard plant SPP model which consumes a maximum power of 25 kW for processing 1000 L of solution. For “electrospinning” process, we selected the Elmarco’s Nanospider Production Line (NS 8S1600U) following the recommendations of the industrial electrospinning equipment by Persano et al. (2013). The Elmarco machine consumed a maximum power of 5 kW for 60 L volume per batch of sample. For “autoclaving” process, we selected the AMSCO Evolution Steam Sterilizer which features a capacity of 141 kg/h with power consumption of 27.23 kW per tonne of products. For “centrifugation” process, we selected the ANDRITZ pusher centrifuge (SZ 800/2) which features power consumption of 40 kW/h (about 3 kW per tonne of product). For “freezer drying” process, we selected the Cuddon Freeze Dryer FD1500 which features a 1500 kg capacity with power consumption of 2kWh/kg. See Table S7 for the energy inventory for the respective equipment.

Table 5 presents the challenges and proposed solutions of the different TENG manufacturing processes at an industrial-scale setup. For “stirring” process, different mixing dynamics (i.e., laminar flow and turbulent flow) occurs when mixing large volume of materials, resulted in non-homogenous mixing. The proposed solution is to mix the large volume of materials in a larger mixing vessel which could produce greater shear force to assist in mixing homogenously. For “electrospinning” process, the challenges include (i) high-capacity production, (ii) high-voltage required for generating multiple liquid jets, (iii) multi-disciplinary knowledge, (iv) lack of control of process parameters, and (v) accuracy and reproducibility of product. The proposed solution is to add salt to increase the quantity of charges to aid in high-capacity production and for generating high voltage for multiple liquid jet, and to employ multiple needle system for tuning the chemical composition and inner material structures for accuracy and control to reproduce products consistently. For “autoclaving” process, it was found that the temperature fluctuations in the autoclave resulted in non-uniform temperature distribution across the product. The proposed solution is to is to use an out-of-autoclave post-processing method to ensure that the product (i.e., aerogel in non-freestanding form) is completely crosslinked to fibrous structure. For “centrifugation” process, the equipment requires constant feeding of materials (a non-stop process) due to its continuous nature and also requires frequent maintenance. The proposed solutions are to develop production plans to forecast the required stock to control the material inventory and to develop preventive maintenance schedule to allocate time to service the equipment. For “freeze drying” process, the temperature fluctuates and causes non-uniform temperature distribution. The duration of the process is typically very long (about 24 h per batch) and also causes high-energy consumption. The proposed solution is to implement and combine new technologies like ultrasound technology or microwave technology to expedite the process to reduce the drying time, energy consumptions, and green-house gas emissions (Jangam et al. 2015; Rybak et al. 2021).

**Table 5:** Challenges and proposed solutions of the different TENG manufacturing process at industrial level

Process	Challenges	Proposed solutions
Stirring	Different mixing dynamics, i.e., production of laminar type flow (liquid undergoing mixing flows in layers which passes over each other following a smooth path and the layers of fluid never interfere with each other) rather than a turbulent flow (the interface between fluidic layers undergo distortion, allowing mixing in both lateral and vertical dimensions) (Azom 2017)	Larger mixing vessels can produce greater shear forces which help to compensate for the lack of turbulent flow
Electrospinning	(i) Production capacity, (ii) High voltage requirement for generating enormous number of liquid jets, (iii) Multi-disciplinary knowledge required for successful manufacturing, (iv) Lack of control over solution and process parameters during production, and (v) Accuracy and reproducibility (Elmarco n.d.)	Salts (made of ions with small radii and high charge density) can be added for increasing the quantity of charges Multiple needle system for tuning the chemical composition and inner structures (Li et al. 2021)
Autoclaving	Temperature fluctuation and non-uniform temperature distribution (Koushyar et al. 2012)	Using out-of-autoclave method to ensure complete cross-linking of aerogel to fibrous structure
Centrifugation	(i) Requires constant feeding of materials to the centrifuge due to continuous nature (Kempf 2010) (ii) High maintenance required	Production planning to forecast the required stocks to control the materials inventory Scheduled preventive maintenance to service the equipment
Freeze drying	Temperature fluctuation and non-uniform temperature distribution (Tsinontides et al. 2004) High energy consumption	Inclusion of new technologies like ultrasound technology or microwave technology in combination to fasten the process, reduce the drying time and consequent energy consumptions and green-house gas emissions (Jangam et al. 2015; Rybak et al. 2021)

#### Environmental impact: laboratory- and industrial-scale setup to manufacture TENG

**Fig. 8:**
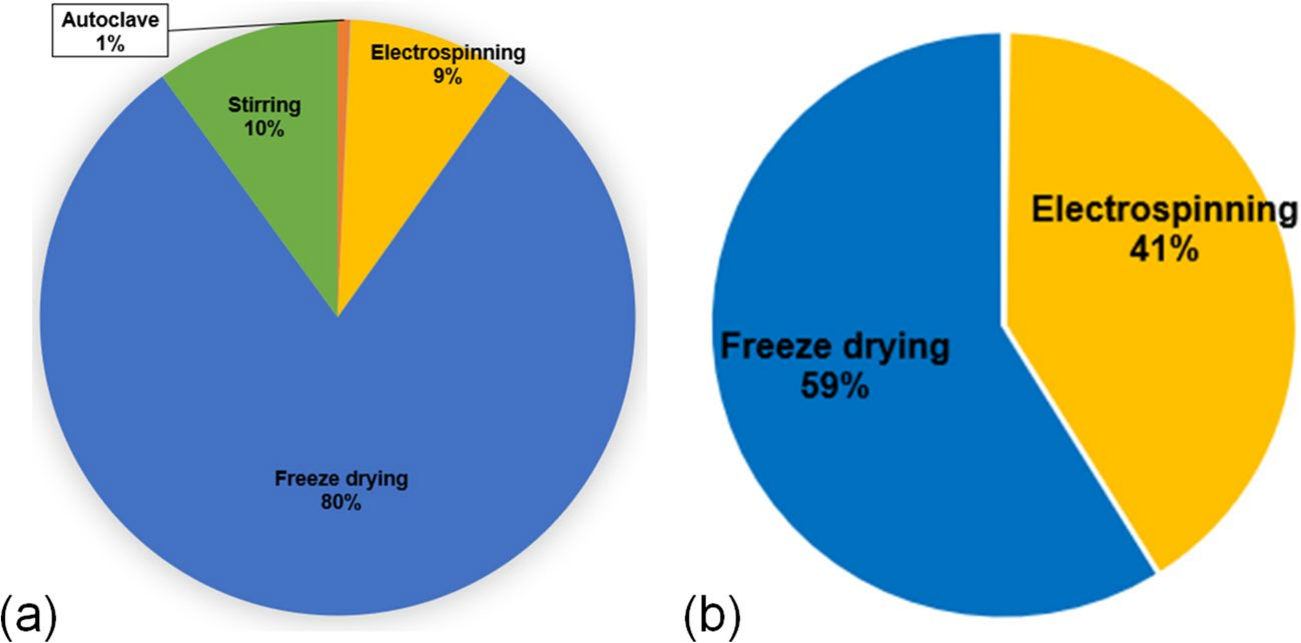
Distribution of carbon footprint for fabricating TENG from PET waste in (**a**) lab-scale setup and (**b**) industrial-scale setup

Figure 8(a) and (b) displays the carbon footprint of TENG manufacturing processes at lab-scale and industrial-scale for producing one ton of PET aerogel, respectively. Carbon footprint was determined by direct greenhouse gas emissions and electricity consumption. Results using the ReCipe method indicate that carbon dioxide emissions are the primary contributor due to low conversion rates and limitations in modeling recycle streams at the lab-scale. Additionally, other emissions (e.g., wastewater and vapor from nanofiber treatment) occur, but upscaling may reduce process emissions through improved CO_2_ conversion rates and recycling options.

In the lab-scale setup, electricity is the primary energy source, and both energy consumption and carbon footprint follow similar patterns. Figure 8(a) indicates that “freeze drying” (~ 80%), “stirring” (~ 10%), and “electrospinning” (~ 9%) processes are the major contributors to the overall carbon footprint. “Freeze drying” contributes the most to carbon dioxide emissions due to its prolonged duration (about 24 h) and high energy consumption.

**Fig. 9:**
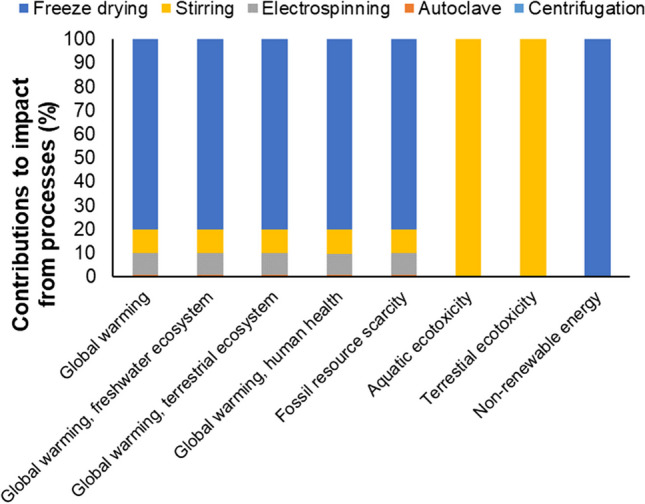
Contributions of environmental impact from the TENG manufacturing processes in lab-scale setup

Figure 9 displays the environmental impact contribution from lab-scale TENG production processes, as evaluated using Impact 2002 + methods. Given that this proposed model is a potential technology for mitigating climate change compared to conventional plastic waste management methods, the focus of the life cycle impact analysis is mainly on assessing global warming impact. The analysis indicated that the freeze-drying process (~ 80%) is the main contributor to global warming, followed by the stirring process (~ 10%) and the electrospinning process (~ 9%). These contributions primarily result from electrical consumption of equipments and machines, leading to carbon dioxide emissions. Additionally, other environmental impacts, such as fossil resource scarcity and non-renewable energy, are mainly linked to equipment/machine energy consumption. The stirring process is the sole contributor to both aquatic and terrestrial ecotoxicity due to its use of trifluoroacetic acid during mixing, which is considered toxic to air, water, and soil in aquatic and terrestrial ecosystems. While these environmental impact results may appear insignificant in the lab-scale setup for TENG manufacturing, potential alternatives will be needed when upscaling manufacturing processes for industrial production of TENG, potentially eliminating the use of trifluoroacetic acid.

Figure 8(b) illustrates the main contributors to the overall carbon footprint after upscaling TENG manufacturing processes for the industrial-scale setup. The industrial setup brings advantages, reducing energy consumption and greenhouse gas emissions. The distribution of the carbon footprint, based on pilot-scale reference models, reveals that existing technologies deployed in commercial settings can benefit from further research for sustainable TENG manufacturing. Nonetheless, it is challenging to depict manufacturing technology innovations in these sectors to achieve a significant reduction in gas emissions and energy demand. The analysis indicates that the primary contributors to global warming are the freeze-drying process (~ 58%) and the electrospinning process (~ 40%). In this context, the contributions from all other manufacturing processes (stirring, autoclaving, and centrifugation) are considered negligible.

#### Comparison between laboratory- and industrial-scale setup to manufacture TENG

When scaling up manufacturing processes from the lab to industrial scale for commercial purposes, production capacity becomes crucial, particularly for high sale volume and versatile applications. These applications include low-voltage uses like wearables and contact tracing devices, as well as high-voltage applications such as plasma generators and electric guns.

In a comparison between lab and industrial setups to produce one ton of PET aerogel for TENG devices, the environmental assessment reveals that the industrial “freeze-drying” process reduces the carbon footprint by approximately 20% compared to lab-scale processes. For other industrial manufacturing processes (stirring, autoclaving, and centrifugation), their carbon footprint contribution is negligible. However, the industrial “electrospinning” process significantly contributes to the carbon footprint. Ongoing research aims to improve electrospinning technologies, given the advantages of employing electrospun nanofibers in various innovative applications, offering commercialization opportunities and potential for nanomaterials (Persano et al. 2013).

It is important to note that in the industrial-scale setup, TENG manufacturing consumes significant amounts of water (about 40,000 L) and ethanol (around 20,000 L). The wastewater and vapors produced during the manufacturing process may impact the environment, considering water withdrawal, treatment, and distribution. Future studies should address these aspects.

### Sensitivity analysis

The details of the results from the sensitivity analysis of the MFA (see Supplementary Information, Tables S18 and S19), and the respective impact categories to the plastic waste and recyclable management processes (see Supplementary Information, Tables S20 to S23), and manufacturing of TENG (see Supplementary Information, Table S24) in Singapore and India are found in the supplementary information.

The results were analyzed based on data quality aspects corresponding to the lowest DQI scores (1, most reliable) and highest DQI score (4, least reliable). A coefficient of variation (CV, see Eq. 1) of 2.3% was calculated when all data quality aspects were reliable (score of 1), while a CV of 103.4% was calculated when all data quality aspects were least reliable (score of 4).

The mass of materials flowing through the different plastic waste and recyclable management processes (Supplementary Information, Tables S18 and S19), respective environmental impact contributed by the respective life cycle process (Supplementary Information, Tables S20 to S23), and TENG manufacturing processes (Supplementary Information, Table S24) varied by 2.3% and 103.4%. The large CV of dataset may affect the analysis of the material mass and environmental assessment. The factors that may contribute to the large CV include (1) types of assumptions that have been made, (2) reliability of data from articles/reports and literature, and (3) unavailability of data. With regard to the MFA, sensitivity analysis was not conducted for the transport distance of the plastics because the uncertainty of the transport distance and the contribution towards the overall impact may be negligible (Van Eygen et al. 2017).

The environmental assessment results represent national averages within the geographical boundaries of Singapore and India. However, it is essential to recognize that these results may not be applicable to every area within these countries. For example, typical urban areas in India, which tend to have better waste management infrastructure, may experience lower levels of mismanaged plastic waste compared to rural areas (Van Eygen et al. 2017).

The dataset used in this study to assess material mass and environmental impact from the life cycle process and TENG manufacturing was derived from government reports, news articles, and literature. Tracking this dataset was challenging, leading to potential underreporting of mismanaged waste and recyclables. Mismanagement includes uncollected plastic waste, illegal dumping, open burning, losses during transportation, illegal imports, and theft. The increase in mismanaged waste can result in a lower proportion of accurately reported collected plastic waste. Additionally, the quantity of plastic rejectables during mechanical recycling varied and had uncertain data.

### Techno-economic analysis for upcycling plastic to manufacture TENG

Table 6 presents the economic values for processing plastic scraps through different treatment processes in Singapore and India. The economic values were calculated based on the costs and revenues of the plastic scrap treatment processes, considering the data obtained from the MFA (Section 3.2), including the amount of PET materials and energy required for processing plastic scraps.

**Table 6:** Economic values of variable operation, fixed operation, and human resources for the PET materials and plastic treatment processes in Singapore and India

Economic parameters	Detail	Country	Value (USD)	Reference
Variable operational costs – material	PET light	Singapore	900 per ton	(2 Lians Pte Ltd 2021)
	PET colored		800 per ton	(2 Lians Pte Ltd 2021)
	Opaque PET		800 per ton	(2 Lians Pte Ltd 2021)
	PET light	India	850 per ton	(IndiaMART 2021)
	PET colored		750 per ton	(IndiaMART 2021)
	Opaque PET		750 per ton	(IndiaMART 2021)
Variable operational costs – electricity	Electricity	Singapore	0.178 per kwh	(GlobalPetrolPrices.com 2021)
	Electricity	India	0.082 per kwh	(GlobalPetrolPrices.com 2021)
Fixed operating expenses	Recycling	Singapore	141 per ton	(RFL 2021)
	Incineration		44 per ton	(National Environment Agency 2022a)
	Landfill		57 per ton	(National Environment Agency 2022a)
	Recycling	India	Undisclosed	_
	Incineration		118 per ton	(Ankur Scientific 2018)
	Landfill		14.8 per ton	(Chhabra et al. 2021)
Investment costs	Recycling	Singapore	110 per ton	(Ong 2021)
	Incineration		0.22 per ton	(National Environment Agency 2022a)
	Landfill		216.2 per ton	(National Environment Agency 2015)
	Recycling	India	Undisclosed	_
	Incineration		Undisclosed	_
	Landfill		0.79 per ton	(Chhabra et al. 2021)
Human resource costs	Recycling	Singapore	30,508 + per year	(ERI Economic Research Institute 2021a)
	Incineration		26,043 + per year	(ERI Economic Research Institute 2021b)
	Landfill		28,275 + per year	(ERI Economic Research Institute 2021c)
	Recycling	India	2738 + per year	(SalaryExpert 2021b)
	Incineration		1882 + per year	(Salary Expert 2021c)
	Landfill		3984 + per year	(SalaryExpert 2021a)

The economic parameters taken into consideration include (1) variable operational costs, (2) fixed operating expenses, (3) investment costs, and (4) human resource costs. The operational cost is a critical aspect to assess the techno-economic viability. The operational costs can be divided into two categories, namely (1) variable and (2) fixed costs. The variable operational costs of materials and electricity vary according to the types of plastic treatment processes. The material cost encompasses the cost of different types of recycled PET granules. PET plastic is naturally transparent and inclusion of colorants increases the challenges in the recycling process, resulting from minimal to no market values for high quantities of colored and opaque recycled PET materials (Sarda et al. 2022).

Fixed operational costs remain stable in the short term for different plastic treatment processes. However, the fixed operating costs of various plastic treatment technologies for processing plastic scraps significantly differ between the two countries.

In Singapore, the cost of plastic recycling was approximately USD$141 per ton. The fixed operating costs for plastic recycling in India were undisclosed and challenging to estimate. For Singapore, plastic recycling was primarily conducted in a neighboring country, Malaysia. Therefore, the fixed operating costs were estimated based on transportation expenses to move plastic scraps from Singapore to Malaysia and the plastic recycling process costs in Malaysia.

In India, the fixed operating costs for recycling were not disclosed and difficult to ascertain due to the significant role of the informal sector in plastic waste and recycling management practices (CSE 2021).

For incineration, operating costs in India were higher than in Singapore. This difference was due to Singapore’s more advanced incineration technology for converting plastic waste to energy (i.e., electricity). In contrast, India has underdeveloped incineration infrastructure, requiring additional processes that result in higher operational costs (Bhawan and Nagar 2013).

Regarding landfilling, operating costs in Singapore were higher than in India. Singapore transported plastic waste to its offshore engineered landfill, while India openly dumped plastic waste in both authorized and unauthorized sites (Central Pollution Control Board 2019; National Environment Agency 2022a).

India did not disclose the investment costs for recycling and incineration infrastructure, as these were managed by both the formal and informal sectors. In Singapore, the investment for recycling depended on the recycling infrastructure in neighboring countries, notably Malaysia. The investment costs for incineration were calculated based on the highest values of investment in incineration infrastructure, notably the Tuas South incineration plant. For landfilling, the investment costs were higher in Singapore compared to India, primarily due to the construction of offshore engineered landfill facilities.

The human resource costs take into account the valuation of human resource. For Singapore, the investment in human resource was much higher as compared to India which may be attributable to higher GDP per capita of the island nation.

Table 7 shows the economic values of the materials, equipment investment, and operational costs for TENG manufacturing in laboratory-scale and industrial-scale setup. The different types of processes for manufacturing the TENG using recycled PET aerogel in laboratory-scale setup have been described in our previous article (Roy et al. 2021). In order to mass manufacture the TENG, we presented the problems, challenges, and the types of equipment for upscaling the manufacturing process from laboratory-scale to industrial-scale setup as described in Section 3.3.1.

**Table 7:** Economic values of materials and equipment investment for TENG manufacturing in laboratory- and industrial-scale setup

Economic parameters	Detail	Lab-scale for producing 25-mg PET aerogel per cycle	Industrial-scale for producing 1-ton PET aerogel per cycle	Remarks
		Value (USD)	Value (USD)	
Material costs	Trifluoroacetic acid	0.06	2348	
	Dichloromethane	0.01	21	
	Ethanol	0.04	16,000	
	Distilled water	0.33	13,200	
	1,3 benzenedisulphonyl azide	0.04	1477	
	Dopamine and Trizma solution	1.31	77,000	
	Polyetherimide	0.044	1760	
Investment costs	Stirring	1005	23,500	
	Electrospinning	10,659	1,100,000	
	Hydrothermal autoclave	1477	35,000	
	Centrifugation	3572	25,000	
	Freeze drying	3540	250,000	
Operational costs—electricity	Stirring	1.18	4.45	Costs in Singapore
		0.55	2.05	Costs in India
	Electrospinning	1.07	5933.10	Costs in Singapore
		0.50	2733.23	Costs in India
	Hydrothermal autoclave	0.81	34.42	Costs in Singapore
		0.37	15.86	Costs in India
	Centrifugation	0.01	1.61	Costs in Singapore
		0.01	0.74	Costs in India
	Freeze drying	9.40	8,544	Costs in Singapore
		4.33	3,936	Costs in India

We derived the economic value of the materials, the investment of equipment, and the operational costs (namely for electricity) to manufacture TENG in laboratory- and industrial-scale setup. The material costs were derived based on the amount of materials required for producing 25 mg and 1 ton of PET aerogel in lab- and industrial-scale setup, respectively. The equipment is based on the lists as described in Table S7 and the costs were obtained from the respective equipment manufacturer and supplier website. The operational costs for electricity were derived based on the electrical consumption of the equipment used to produce the PET aerogel in Singapore and India.

The material costs for industrial production of 1-ton PET aerogel were directly proportional to the amount of materials required to produce 25 mg of PET aerogel in a lab-scale setup. For investment costs of equipment, it was observed that the costs of the industrial-scale equipment (total equipment costs ≈ USD$1,433,500) were 70 times higher than the lab-scale equipment (total costs ≈ USD$20,253). However, the industrial-scale equipment could produce 36 million times more PET aerogel at a single cycle as compared to the lab-scale equipment. For the operational costs (namely electricity) to manufacture 1 ton of PET aerogel, the costs of electricity for using the industrial-scale equipment (total electricity costs ≈ USD$14,518 in Singapore and USD$6688 in India) were much cheaper than operating multiple cycles of PET aerogel manufacturing process using lab-scale equipment (total electricity costs ≈ USD$453 million in Singapore and USD$210 million in India). With regard to the mass manufacturing of PET aerogels, to produce a mass of 1 ton of PET aerogel, the use of the industrial-scale equipment could significantly reduce the production time compared to the lab-scale equipment.

The analysis concluded that the material costs, equipment investment costs, and the operational costs increase when the scale of manufacturing of PET aerogel expanded from laboratory to industrial scale setup. However, for manufacturing high volume of PET aerogel (i.e., 1 ton of PET aerogel), the industrial-scale manufacturing setup is much more cost-saving for long-term manufacturing operation and time-efficient for producing high volume of products than the laboratory-scale manufacturing setup. Furthermore, regardless of lab- or industrial-scale setup, it was observed that the operational costs (specifically on electricity) for manufacturing PET aerogel in Singapore were about 2.2 times higher than in India. This is due to the use of natural gas for electricity production in Singapore that resulted in higher electricity pricing as compared to India which mainly relied on burning coal. The techno-economic analysis showed that the industrial-scale setup for manufacturing PET aerogel was promising and could increase the recycling rate of plastic in both India and Singapore. Furthermore, the TENG technology could potentially improve the knowledge of the people in the countries to understand the importance of reduce, reuse, and recycle of plastics; technology implementation could create more job opportunities (such as new hire for operators, technicians, engineers, researchers, etc.); and technology advancement could enhance the knowledge of the people which may help to improve their wages.

## Conclusions and future perspectives

The comparative study assesses plastics’ lifecycle in TENG manufacturing in India and Singapore. The material flow analysis (MFA) reveals similar processes but distinct waste distribution and treatment methods. Environmental impact analysis indicates that Singapore has higher emissions in waste collection, while India’s recycling results in a lower net environmental impact. TENG manufacturing, scaled from lab to industrial settings, shows a 20% reduction in carbon footprint in the “freeze-drying” process. Key challenges in TENG manufacturing include equipment-related issues. Techno-economic analysis indicates increased costs in scaling PET aerogel manufacturing, but industrial setups prove cost-effective for high-volume production.

The study suggests TENG from recycled plastics as a promising solution for plastic waste. The combined MFA and LCA provide insights into waste management systems, guiding researchers and policymakers. Prospective LCA for TENG emphasizes environmental, economic, and social aspects, acknowledging uncertainties. To ensure circular resource flow, future considerations should include end-of-life options, remanufacturing, and lifetime assessments in circularity studies. Stakeholders should focus on design choices’ environmental impacts for market penetration and sustainable outcomes.

### Supplementary Information

Below is the link to the electronic supplementary material.

Supplementary file1 (DOCX 87 KB)

## Data Availability

The data are available in the Supplementary Information document.

## Abbreviations

*CPCB*: Central Pollution Control Board
*CV*: Coefficient of variation
*DQI*: Data quality indicators
*EoL*: End-of-life
*LCA*: Life cycle assessment
*LCI*: Life cycle impact
*MFA*: Material flow analysis
*MRF*: Materials recovery facilities
*NEA*: National Environmental Agency
*PET*: Polyethylene terephthalate
*PWM*: Plastic waste management
*TENG*: Triboelectric nanogenerator
*WTE*: Waste-to-energy
