# Supplementary material for: Gate-to-grave assessment of plastic from recycling to manufacturing of TENG: a comparison between India and Singapore
Source: Environ Sci Pollut Res Int. 2024 Jun 15;31(30):42698–718. doi: 10.1007/s11356-024-33867-w (PMC11222216; doi:10.1007/s11356-024-33867-w)
Supplement: Supplementary file 1 — Supplementary file1 (DOCX 87 KB) [file 11356_2024_33867_MOESM1_ESM.docx]

**Gate-to-grave assessment of plastic from recycling to manufacturing: A comparison between India and Singapore**

Shreya Sharma, Wei Liang Lai, Sunanda Roy, Pradip Kumar Maji, Seeram Ramakrishna, Kheng Lim Goh

Table S1. Input-output data for mechanical recycling in Singapore and India. The data shown were derived based on 1kg of plastic waste.

| **Singapore** | |
| --- | --- |
| **Energy input (all units in kWh)** | |
| Natural gas | 9.10x10^8^ |
| Electricity | 3.82x10^8^ |
| **Process emission, Output (all units in kg)** | |
| Carbon dioxide | 7.56x10^8^ |
| **India** | |
| **Energy input (all units in kWh)** | |
| Electricity | 2.16x10^9^ |
| **Process emission, Output (all units in kg)** | |
| Carbon dioxide | 2.77x10^8^ |
| Carbon monoxide | 9.21x10^6^ |
| Nitrogen oxides | 1.75x10^5^ |
| Particulates | 2.36x10^5^ |
| Sulphur oxides | 1.78x10^4^ |

Table S2. Input-output data for WTE incineration in Singapore and India. The data shown were derived based on 1kg of plastic waste.

| **Singapore** | |
| --- | --- |
| **Energy input (all units in kWh)** | |
| Natural gas | 2.25x10^10^ |
| Electricity | 5.82x10^10^ |
| **Process emission, Output (all units in kg)** | |
| Carbon dioxide | 2.12x10^17^ |
| Dioxin | 3.00x10^14^ |
| Furan | 3.00x10^14^ |
| Hydrogen chloride | 127.30 |
| Mercury compounds | 3.00x10^9^ |
| Nitrogen oxides | 1.74x10^12^ |
| Particulates | 2.08x10^6^ |
| Carbon dioxide | 2.12x10^17^ |
| **India** | |
| **Energy input (all units in kWh)** | |
| Electricity | 8.06x10^7^ |
| **Process emission, Output (all units in kg)** | |
| Carbon dioxide | 2.40x10^8^ |
| Carbon monoxide | 4.07x10^7^ |
| Ethylene | 1.52x10^6^ |
| Particulates | 9.47x10^6^ |
| Hydrocarbons | 5.18x10^6^ |

Table S3. Output data for plastic waste disposal in landfills of Singapore and India. The data shown were derived based on 1kg of plastic waste.

| **Singapore** | |
| --- | --- |
| **Process emission, Output (all units in kg)** | |
| Carbon dioxide | 1.76x10^8^ |
| Hydrogen chloride | 233792 |
| Carbon monoxide | 1.41x10^5^ |
| Nitrogen oxides | 1.38x10^5^ |
| Hydrogen fluoride | 46.68 |
| Hydrogen sulfide | 3.28x10^5^ |
| Metals | 918.53 |
| Methane | 4.92x10^7^ |
| Sulphur oxides | 3.28x10^5^ |
| **India** | |
| **Process emission, Output (all units in kg)** | |
| Carbon dioxide | 9.99x10^10^ |
| Sulphur oxides | 3.28x10^8^ |
|  |  |

Table S4. Output data for cement-kiln co-processing in India. The data shown were derived based on 1kg of plastic waste.

| **India** | |
| --- | --- |
| **Process emission, Output (all units in kg)** | |
| Carbon dioxide | 1.23x10^8^ |
| Dioxin and Furan | 1.25x10^7^ |
| Carbon monoxide | 8.06x10^4^ |
| Mercury compounds | 140.79 |
| Nitrogen oxides | 1.99x10^5^ |
| Sulphur oxides | 7.17x10^4^ |
| Hydrocarbons | 1.63x10^4^ |

Table S5. Input-Output data for local transportation in Singapore. The data shown were derived based on 1kg of plastic waste.

| **Singapore** | |
| --- | --- |
| **Energy input (all units in kWh)** | |
| Natural gas | 5.70x10^6^ |
| **Process emission, Output (all units in kg)** | |
| Carbon monoxide | 8544.38 |
| Hydrocarbons | 740.51 |
| Nitrogen oxides | 2278.50 |
| Non-methane hydrocarbons | 911.40 |
| Particulates | 56.96 |

Table S6. Input-Output data for local transportation in India. The data shown were derived based on 1kg of plastic waste.

|  | **Used Bottles to Traders/MRF** | **Crushed, Bundled Bottles to PWM sites** |
| --- | --- | --- |
| **Energy input (all units in kWh)** | | |
| Natural gas | 8.84x10^6^ | 23.7x10^6^ |
| **Process emission, Output (all units in kg)** | | |
| Carbon monoxide | 2.01x10^4^ | 9.47x10^4^ |
| Mercury compounds |  | 140.79 |
| Nitrogen oxides | 7.25x10^2^ | 3.46x10^6^ |
| Particulates | 39.8 | 236.7792 |
| Methane |  | 1.18x10^5^ |
| Sulphur oxides |  | 7.17x10^4^ |
| Hydrocarbons | 1414.36 | 3.79x10^4^ |

Table S7. Input-output data for manufacturing of TENG using (a) laboratory-scale equipment and (b) industrial-scale equipment.

| 1. **Laboratory-scale, by per 25 mg of aerogel** | | | | |
| --- | --- | --- | --- | --- |
| **Processes** | **Equipment model** | **Power (W)** | **Time (hrs)** | **Electricity (kWh)** |
| Magnetic stirring | IKA | 550 | 12 | 6.6 |
| Electrospinning | MTI  (MSK-NFES-3) | 3000 | 2 | 6 |
| Hydrothermal autoclave | Goldbrunn (450) | 450 | 10 | 4.5 |
| Centrifugation | bioSan  (LMC-3000) | 110 | 0.25 | 0.0275 |
| Freeze drying | Parisa Technology (Lyophilizer Freezer Dryer) | 2200 | 24 | 52.8 |
| 1. **Industrial-scale, by per ton of aerogel** | | | | |
| **Energy input** | **Equipment model** | **Power (kW)** | **Time (hrs)** | **Electricity (kWh)** |
| Stirring | IKA (SPP4000) | 25 | 1 | 25 |
| Electrospinning | Elmarco (NS8S1600U) | 83 | 400 | 33332 |
| Hydrothermal autoclave | AMSCO (HC1500) | 27 | 7 | 193 |
| Centrifugation | ANDRITZ (SZ 800/2) | 9 | 1 | 9 |
| Freeze drying | Cuddon (FD1500) | 2000 | 24 | 48000 |

Table S8. Data for the amount of materials used to treat PET scraps for making aerogels in lab scale and industrial scale.

| **Material input (ml)** |  | **Lab-scale for making 25 mg aerogel (ml)** | **Upscale for industrial setting for making 1 ton of aerogel (ml)** |  |  |  |
| --- | --- | --- | --- | --- | --- | --- |
| Trifluoroacetic acid |  | 0.1 | 4000 |  |  |  |
| Dichloromethane |  | 0.3 | 12000 |  |  |  |
| Ethanol |  | 500* | 20x10^6^ |  |  |  |
| Distilled water |  | 1000* | 40x10^6^ |  |  |  |
| 1, 3 benzenedisulphonyl azide |  | 0.01 | 400 |  |  |  |
| Dopamine and trizma solution |  | Trizma HCl:1.011g; Trizma base: 11.337g | Trizma HCl: 40.44x10^3^ kg; Trizma base: 453.48x10^3^ kg |  |  |  |
| Polyetherimide |  | 0.1 | 4000 |  |  |  |
| **Process emissions** |  | **Average (world)** | **India** | | **Singapore** | |
|  |  |  | **Lab scale** | **Industrial scale** | **Lab scale** | **Industrial scale** |
| Co2 |  | 0.475 kg/kwh  (IEA, n.d.) | 0.95 kg/kwh | _ | 0.408 kg/kwh | _ |
| Wastewater |  | 1000ml | 1000ml | 30000000ml | 1000ml | 30000000ml |

Table S9. Assumptions make in MFA and LCA.

| **Assumption** |  | **Ref.** |
| --- | --- | --- |
| **MFA** |  |  |
| **Singapore** |  |  |
| Incineration process | - The incineration process reduces the plastic waste mass by about 90%, therefore, the residues collected is assumed to be 10% of the total mass of plastic waste. | (National Environment Agency, 2020) |
| **India** |  |  |
| Incineration process | - The incineration process reduces the plastic waste mass by about 90%, therefore, the residues collected is assumed to be 10% of the total mass of plastic waste. | (Central Pollution Control Board, n.d.) |
|  |  |  |
| **LCA** |  |  |
| **Singapore** | - NIL |  |
| **India** | - No residue is generated post cement-kiln co processing of plastics, therefore, we assume 100% conversion rate of plastic into value added products like oil via cement-kiln co-processing | (M. of E. F. and C. C. G. of I. Central Pollution Control Board, n.d.) |
|  |  |  |
| **Manufacturing of TENG** |  |  |
| Manufacturing at lab scale | For producing 23 grams of PET aerogel;   - Distilled water in use: 1000ml (All solutions are prepared in 100 ml distilled water each and remaining water is used in washing.) - Ethanol in use: 500 ml |  |
| Manufacturing at industrial scale | For upscaling to produce 1 ton of PET aerogel;   - The amount of chemicals and solvent used to treat the PET scraps were upscaled by direct proportion of the PET aerogel weight. (1 mg of PET aerogel make in lab to 1 ton of PET aerogel.) |  |
|  |  |  |
| **Costs Analysis** |  |  |
| **Singapore** |  |  |
| Transportation | - Transportation costs (SGD80 per ton) from the waste disposal location-to-MRF and MRF-to-WTE plant were assumed to be the same, and the truck used were also similar. - Most of the plastic recyclable were sent to neighbouring country for recycling (i.e. Johor, Malaysia). Assuming that the distance from MRF (V1 Recycle Pte Ltd) to recycling plant (Moldex Plastic Recycling Sdn Bhd) is about 64 km. The transportation costs for 6 tons of plastic recyclable per trip is about SGD200 (based on rental of 24 ft truck). |  |
| **India** | - Transportation from waste collection points to MRF is heavily reliant on the informal sector utilizing range of man-powered vehicles such as pushcart, tri-cycle, electronic rickshaw, etc. and therefore the transportation cost is considered negligible and not taken into account. - Transportation costs from MRF to different waste disposal sites were assumed to be the same, and the truck used were also similar. | (Central Public Health and Environmental Engineering Organisation (CPHEEO), 2020) |

Table S10. Environmental impact contributed by the respective plastic scrap/recyclable processes in Singapore. The magnitude of the contribution to environmental impact by the plastic management processes were derived using ReCiPe midpoint (H) method.

| **Processes** | **Bundled bottles to waste (kg)** | **Used bottle to traders/MRF (kg)** | **Mechanical recycling (kg)** | **Incineration (kg)** | **Landfill (kg)** |
| --- | --- | --- | --- | --- | --- |
| Fine particulate matter formation | -3.37E-14 | 2.51E+02 |  | 17.17E-03 | 52.83E+03 |
| Fossil resource scarcity |  |  | 115.12E+07 | 1.59E-07 |  |
| Freshwater ecotoxicity |  |  |  | 3.50E-08 |  |
| Global warming |  |  | 121.52E+08 | 19.04 | 64.27E+08 |
| Human carcinogenic toxicity |  |  |  | 2.55 |  |
| Human non-carcinogenic toxicity |  |  |  | 0.95 |  |
| Marine ecotoxicity |  |  |  | 6.83E-07 |  |
| Ozone formation, human health | -3.07E-13 | 22.79E+02 |  | 156.06E-03 | 48.03E+04 |
| Ozone formation, terrestrial ecosystems | -3.07E-13 | 22.79E+02 |  | 156.06E-03 | 48.03E+04 |
| Terrestrial acidification | -1.10E-13 | 8.20E+02 |  | 56.18E-03 | 17.30E+04 |
| Terrestrial ecotoxicity |  |  |  | 6.59E-05 |  |

Table S11. Environmental impact contributed by the respective plastic scrap/recyclable processes in Singapore. The magnitude of the contribution to environmental impact by the plastic management processes were derived using ReCiPe endpoint (H) method.

| **Processes** | **Units** | **Used bottle to Traders/MRF** | **Bundled PET Bottles to Waste** | **Mechanical recycling** | **Incineration** | **Landfill** |
| --- | --- | --- | --- | --- | --- | --- |
| **Impact categories** |  |  |  |  |  |  |
| Fine particulate matter formation | DALY-disability adjusted loss of life years | 157.67E-03 | -2.12E-17 |  | 1.08E-05 | 33.23 |
| Fossil resource scarcity | USD 2013 |  |  | 4.14E08 | 5.71E-08 |  |
| Freshwater ecotoxicity | species.yr |  |  |  | 2.42E-17 |  |
| Global warming, freshwater ecosystems | species.yr |  |  | 9.30E-04 | 1.46E-12 | 4.92E-4 |
| Global warming, human health | DALY |  |  | 1.13E04 | 1.77E-05 | 5.97E03 |
| Global warming, terrestrial ecosystems | species.yr |  |  | 34.03 | 5.33E-08 | 18.00 |
| Human carcinogenic toxicity | DALY |  |  |  | 8.47E-06 |  |
| Human non-carcinogenic toxicity | DALY |  |  |  | 2.17E-07 |  |
| Marine ecotoxicity | species.yr |  |  |  | 7.18E-17 |  |
| Ozone formation, human health | DALY | 2.07E-03 | -2.79E-19 |  | 1.42E-07 | 43.71E-02 |
| Ozone formation, terrestrial ecosystems | species.yr | 2.94E-04 | -3.96E-20 |  | 2.01E-08 | 61.96E-03 |
| Terrestrial acidification | species.yr | 1.74E-04 | -2.34E-20 |  | 1.19E-08 | 36.65E-03 |
| Terrestrial ecotoxicity | species.yr |  |  |  | 7.50E-16 |  |

Table S12. Gas emission by the respective plastic scrap/recyclable processes in Singapore. The magnitude of the gas emission by the plastic management processes were derived using ReCiPe midpoint (H) method.

| **Emissions** | **Bundled bottles to waste (kg)** | **Used bottle to traders/MRF (kg)** | **Mechanical recycling (kg)** | **Incineration (kg)** | **Landfill (kg)** |
| --- | --- | --- | --- | --- | --- |
| Carbon dioxide |  |  | 121.52E+08 | 19.04 | 613.39E+06 |
| Carbon monoxide | -1.15E-12 | 8544.38 |  |  | 491.87E+03 |
| Dioxins |  |  |  | 26.88E-03 |  |
| Furan |  |  |  | 26.88E-03 |  |
| Hydrogen chloride |  |  |  | 1.14E-14 | 813.03 |
| Hydrogen fluoride |  |  |  |  | 162.32 |
| Hydrocarbons | -9.97E-14 | 740.51 |  |  |  |
| Hydrogen sulfide |  |  |  |  | 114.00E+04 |
| Mercury compounds |  |  |  | 2.69E-07 |  |
| Metals |  |  |  |  | 3194.24 |
| Methane |  |  |  |  | 171.00E+06 |
| Nitrogen oxides | -3.07E-13 | 2278.5 |  | 156.06E-03 | 480.30E+03 |
| Nonmethane hydrocarbons | -1.23E-13 | 911.4 |  |  |  |
| Particulates, < 10 um | -7.67E-15 | 56.96 |  | 1.87E-10 |  |
| Sulphur oxides |  |  |  |  | 114.00E+04 |

Table S13. Environmental impact contributed by the respective plastic scrap/recyclable processes in Singapore. The magnitude of the contribution to environmental impact by the plastic management processes were derived using IMPACT 2002+ method.

| **Processes** | **Units** | **Bundled PET Bottles to Waste** | **Used bottle to Traders/MRF** | **Mechanical recycling** | **Incineration** | **Landfill** |
| --- | --- | --- | --- | --- | --- | --- |
| **Impact categories** |  |  |  |  |  |  |
| Aquatic acidification | kg | -2.15E-13 | 1.59E+03 |  | 10.92E-02 | 24.79E+05 |
| Carcinogens | kg |  |  |  | 20.21E-05 |  |
| Global warming | kg | -1.81E-12 | 13.41E+03 | 121.52E+08 | 19.04 | 19.14E+08 |
| Non-carcinogens | kg |  |  |  | 6.10E-06 | 34.74E+04 |
| Non-renewable energy | MJ | -2.76E-09 | 20.51E+06 | 587.35E+08 | 2.61E-05 |  |
| Respiratory inorganics | kg | -4.32E-14 | 320.54 |  | 19.86E-03 | 61.13E+03 |
| Respiratory organics | kg |  |  |  |  | 10.28E+05 |
| Terrestrial acidification/nutrition | kg | -1.68E-12 | 12.50E+03 |  | 85.65E-02 | 26.36E+05 |

Table S14. Environmental impact contributed by the respective plastic scrap/recyclable processes in India. The magnitude of the contribution to environmental impact by the plastic management processes were derived using ReCiPe midpoint (H) method.

| **Processes** | **Used bottle to Traders/MRF (kg)** | **Crushed, bundle PET bottles to PWM sites (kg)** | **Incineration**  **(kg)** | **Mechanical recycling**  **(kg)** | **Cement Kiln Co-processing**  **(kg)** | **Landfill**  **(kg)** |
| --- | --- | --- | --- | --- | --- | --- |
| **Impact categories** |  |  |  |  |  |  |
| Fine particulate matter formation | 132.89 | 3.23E-11 |  | -7.42E-12 | -88.49E+04 |  |
| Fossil resource scarcity |  |  |  |  |  |  |
| Freshwater ecotoxicity | -3278.43 |  |  |  | -32.78E+02 |  |
| Gloal warming |  | 3.42E-10 | -2.03E-05 | -8.79E-08 |  | 4.89E+12 |
| Human carcinogenic toxicity |  |  |  |  | -22.59E+04 |  |
| Human non-carcinogenic toxicity |  |  |  |  | -80.50E+06 |  |
| Marine ecotoxicity |  |  |  |  | -77.90E+04 |  |
| Ozone formation, human health | 1208.10 | 2.94E-10 | -4.67E-08 | -5.56E-11 | -45.86E+05 |  |
| Ozone formation, terrestial ecosystems | 1208.10 | 2.94E-10 | -7.52E-08 | -5.56E-11 | -45.86E+05 |  |
| Terrestial acidification | 434.92 | 1.06E-10 |  | -2.46E-11 | -29.74E+05 |  |
| Terrestial ecotoxicity |  |  |  |  |  |  |

Table S15. Environmental impact contributed by the respective plastic scrap/recyclable processes in India. The magnitude of the contribution to environmental impact by the plastic management processes were derived using ReCiPe endpoint (H) method.

| **Processes** | **Units** | **Used bottle to Traders/MRF** | **Bundled PET Bottles to PWM sites** | **Incineration** | **Mechanical recycling** | **Cement kiln co-processing** | **Landfill** |
| --- | --- | --- | --- | --- | --- | --- | --- |
| **Impact categories** |  |  |  |  |  |  |  |
| Fine particulate matter formation | DALY-disability adjusted loss of life years | 83.60E-03 | 2.03E-14 |  | -4.67E-15 | -557.17 |  |
| Fossil resource scarcity | USD 2013 |  |  |  |  |  |  |
| Freshwater ecotoxicity | species.yr |  |  |  |  | -2.27E-06 |  |
| Global warming, freshwater ecosystems | species.yr |  | 2.62E-23 | -1.56E-18 | -6.72E-21 |  | 37.42E-02 |
| Global warming, human health | DALY |  | 3.18E-16 | -1.89E-11 | -8.16E-14 |  | 45.48E05 |
| Global warming, terrestrial ecosystems | species.yr |  | 9.58E-19 | -5.70E-14 | -2.46E-16 |  | 1.37E04 |
| Human carcinogenic toxicity | DALY |  |  |  |  | -74.98E-02 |  |
| Human non-carcinogenic toxicity | DALY |  |  |  |  | -18.34 |  |
| Marine ecotoxicity | species.yr |  |  |  |  | -8.18E-05 |  |
| Ozone formation, human health | DALY | 10.99E-04 | 2.67E-16 | -4.24E-14 | -5.06E-17 | -4.17 |  |
| Ozone formation, terrestrial ecosystems | species.yr | 1.56E-04 | 3.79E-17 | -9.69E-15 | -7.18E-18 | -59.15E-02 |  |
| Terrestrial acidification | species.yr | 9.22E-05 | 2.24E-17 |  | -5.21E-18 | -63.11E-02 |  |
| Terrestrial ecotoxicity | species.yr |  |  |  |  |  |  |

Table S16. Gas emission by the respective plastic scrap/recyclable processes in India. The magnitude of the gas emission by the plastic management processes were derived using ReCiPe midpoint (H) method.

| **Processes** | **Used bottle to Traders/MRF (kg)** | **Crushed, bundle PET bottles to PWM sites (kg)** | **Incineration**  **(kg)** | **Mechanical recycling**  **(kg)** | **Cement Kiln Co-processing**  **(kg)** | **Landfill**  **(kg)** |
| --- | --- | --- | --- | --- | --- | --- |
| **Emissions** |  |  |  |  |  |  |
| Carbon monoxide | 33443.74 | 8.05E-12 | -3.45E-06 | -2.92E-09 | -18.57E+05 |  |
| Carbon dioxide |  |  | -2.04E-05 | -8.79E-08 | -28.33E+08 | 2.50E+11 |
| Dioxin and furans |  |  |  |  | -2.89E-06 |  |
| Benzopyrene |  |  | -1.07E-08 |  |  |  |
| Mercury compounds |  |  |  |  | -3245.97 |  |
| Nitrogn oxides | 1208.10 | 2.94E-10 |  | -5.56E-11 | -45.86E+05 |  |
| Particulates | 66.30 | 2.01E-14 | -8.03E-07 | -7.48E-11 |  |  |
| Hydrocarbons | 2357.27 | 3.22E-12 | -4.39E-07 |  | -37.67E+04 |  |
| Metals |  |  |  |  |  |  |
| Methane |  | 1.01E-11 |  |  |  | 1.37E+11 |
| Sulphur oxides |  |  |  | -5.66E-12 | -16.54E+05 |  |
| Ethylene |  |  | -1.28E-07 |  |  |  |
| Hydrocarbons |  |  |  |  |  |  |

Table S17. Environmental impact contributed by the respective plastic scrap/recyclable processes in India. The magnitude of the contribution to environmental impact by the plastic management processes were derived using IMPACT 2002+ method.

| **Processes** | **Used bottle to Traders/MRF (kg)** | **Crushed, Bundled PET bottles to PWM sites (kg)** | **Mechanical recycling (kg)** | **Incineration (kg)** | **Cement kiln co-processing (kg)** | **Landfill (kg)** |
| --- | --- | --- | --- | --- | --- | --- |
| Aquatic ecotoxicity |  |  | -4.35E-11 | -2.38E-10 |  |  |
| Aquatic acidification | 845.67 | 2.06E-10 |  |  |  |  |
| Carcinogens |  |  |  |  |  |  |
| Global warming | 52506.68 | 8.91E-11 | -9.25E-08 | -2.58E-05 |  | 1.29E+12 |
| Respiratory inorganics |  | 3.74E-11 |  | -4.30E-07 |  |  |
| Respiratory organics | 189.29 | 6.05E-14 | -4.75E-11 | -1.28E-07 |  | 82.08E+07 |
| Terrestrial acidification/nutrient | 6630.04 | 1.61E-09 | -3.10E-10 |  |  |  |
| Non-carcinogens |  |  |  |  |  |  |

Table S18. Base results and standard deviation of material flow through different processes in Singapore. The smallest score value of data quality indicator of 1 were selected for all quality aspects to derive for the standard deviations (C.V.= 2.3 %), and the largest score value of data quality indicator of 4 were selected for all quality aspects to derive for the standard deviations (C.V.= 103.5 %).

| **Material flow** | **Mass (Tons/Annum)** | **Standard deviation** | | **Processes** | **Mass (Tons/Annum)** | **Standard deviation** | |
| --- | --- | --- | --- | --- | --- | --- | --- |
|  |  | **DQI = 1**  **(CV = 2.3%)** | **DQI = 4**  **(CV = 103.4%)** |  |  | **DQI = 1**  **(CV = 2.3%)** | **DQI = 4**  **(CV = 103.4%)** |
| F1: Raw materials  (i.e. crude oil) | Undisclosed | - | - | P1: Processing  (Crude oil to plastic materials) | Undisclosed | - | - |
| F2: Intermediate product  (i.e. plastic type) | Undisclosed  (17.76 billion pieces) | - | - | P2: Plastic production  (Plastic materials to goods/products) | Undisclosed | - | - |
| F3: Imported virgin plastic goods/products | +6,000 | 138 | 6,204 | P3: Waste generation  (Plastic goods/ products to waste) | 0 | 0 | 0 |
| F4: Plastic goods/ products | Undisclosed | - | - | P4: MRF  (Waste to different waste treatment) | -6,000 | 138 | 6,204 |
| F5: Plastic waste collection | 868,000 | 19,964 | 897,512 | P5: Mechanical recycling  (Waste to recycled plastics) | 0 | 0 | 0 |
| F6: Recyclables | 42,000 | 966 | 43,428 | P6: Incineration  (Waste to residue) | 0 | 0 | 0 |
| F7: Recycled plastics | 36,000 | 828 | 37,224 | P7: Landfill  (Residue to landfill) | -3,000 | 69 | 3,102 |
| F8: Rejectables from mechanical recycling | 6,000 | 138 | 6,204 | - Undisclosed represents mass data not available or found. - Values with positive sign (+) represents the estimated mass that could be contributed to the material flow. - Values with negative sign (-) represents the estimated mass required to be contributed to a material flow to balance the process. - The zero value of mass represents that the process system is balanced. | | | |
| F9: Incinerable plastic waste | 832,000 | 19,136 | 860,288 |  |  |  |  |
| F10: Residue from incinerated plastic waste | 83,800  (Mass reduction after incineration) | 1,927 | 86,649 |  |  |  |  |
| F11: Non-incinerable plastic waste | +3,000 | 69 | 3,102 |  |  |  |  |
| F12: Total plastic waste in landfill | 86,800 | 1,996 | 89,751 |  |  |  |  |

Table S19. Base results and standard deviation of material flow through different processes in India. The smallest score value of data quality indicator of 1 were selected for all quality aspects to derive for the standard deviations (C.V.= 2.3 %), and the largest score value of data quality indicator of 4 were selected for all quality aspects to derive for the standard deviations (C.V.= 103.5 %).

| **Material flow** | **Mass (Tons/Annum)** | **Standard deviation** | | **Processes** | **Mass (Tons/Annum)** | **Standard deviation** | |
| --- | --- | --- | --- | --- | --- | --- | --- |
|  |  | **DQI = 1**  **(CV = 2.3%)** | **DQI = 4**  **(CV = 103.4%)** |  |  | **DQI = 1**  **(CV = 2.3%)** | **DQI = 4**  **(CV = 103.4%)** |
| F1: Raw materials  (i.e. crude oil) | Undisclosed | - | - | P1: Processing  (Crude oil to plastic materials) | Undisclosed | - | - |
| F2: Intermediate product  (i.e. plastic type) | 15,120,000 | 347,760 | 15,634,080 | P2: Plastic production  (Plastic materials to goods/products) | -6,350,400 | 146,059 | 6,566,314 |
| F3: Imported virgin plastic goods/products | +6,350,400 | 146,059 | 6,566,314 | P3: Waste generation  (Plastic goods/ products to waste) | 0 | 0 | 0 |
| F4: Plastic goods/ products | 8,769,600 | 201,701 | 9,067,766 | P4: MRF  (Waste to different waste treatment) | 0 | 0 | 0 |
| F5: Uncollected plastic waste | 3,507,840 | 80,680 | 3,627,107 | P5: Mechanical recycling  (Waste to recycled plastics) | 0 | 0 | 0 |
| F6: Waste collection | 5,261,760 | 121,020 | 544,060 | P6: Cement-kiln co processing | 0 | 0 | 0 |
| F7: Recyclable | 3683,232 | 84,714 | 3,808,462 | P7: Incineration | -126,282 | 2,904 | 130,576 |
| F8: Recycled plastics | 3683,232 | 84,714 | 3,808,462 | P8: Open dumping/burning/landfill | -4,818,018 | 110,814 | 4,981,831 |
| F9: Incinerable plastic waste to cement-kiln co processsing | 142,068 | 3,268 | 146,898 | - Undisclosed represents mass data not available or found. - Values with positive sign (+) represents the estimated mass that could be contributed to the material flow. - Values with negative sign (-) represents the estimated mass required to be contributed to a material flow to balance the process.   The zero value of mass represents that the process system is balanced. | | | |
| F10: Plastic waste to value added products like fuel etc. | 142,068 | 3,268 | 146,898 |  |  |  |  |
| F11: Incinerable plastic waste | +126,282 | 2,904 | 130,576 |  |  |  |  |
| F12: Residue from incineration plastic waste | 12,628  (Mass reduction after incineration) | 290 | 13,058 |  |  |  |  |
| F13: Non-Incinerable plastic waste | 1,310,178 | 30,134 | 1,354,724 |  |  |  |  |
| F14: End of life | +4,818,018 | 110,814 | 4,981,831 |  |  |  |  |

Table S20. Base results and standard deviation of different impact categories contributed by the respective processes in Singapore derived using ReCiPe midpoint (H) method. The smallest score value of data quality indicator of 1 were selected for all quality aspects to derive for the standard deviations (C.V.= 2.3 %).

| **Processes (Standard deviation @ DQI =1)** | **Bundled PET Bottles to Waste** | | **Used bottle to Traders/MRF** | | **Mechanical recycling** | | **Incineration** | | **Landfill** | |
| --- | --- | --- | --- | --- | --- | --- | --- | --- | --- | --- |
|  | **Base result** | **Standard deviation** | **Base result** | **Standard deviation** | **Base result** | **Standard deviation** | **Base result** | **Standard deviation** | **Base result** | **Standard deviation** |
| Fine particulate matter formation | -3.37E-14 | 7.76E-16 | 250.64 | 5.77 |  |  | 0.0172 | 0.000395 | 52832.27 | 1215.14 |
| Fossil resource scarcity |  |  |  |  | 1151957520 | 26495022.96 | 1.59E-07 | 3.66E-09 |  |  |
| Freshwater ecotoxicity |  |  |  |  |  |  | 3.50E-08 | 8.04E-10 |  |  |
| Global warming |  |  |  |  | 12152000000 | 279496000 | 19.04 | 0.438 | 6427250667 | 147826765.3 |
| Human carcinogenic toxicity |  |  |  |  |  |  | 2.55 | 0.0587 |  | 0 |
| Human non-carcinogenic toxicity |  |  |  |  |  |  | 0.95 | 0.0219 |  |  |
| Marine ecotoxicity |  |  |  |  |  |  | 6.83E-07 | 1.57E-08 |  |  |
| Ozone formation, human health | -3.07E-13 | 7.06E-15 | 2278.5 | 52.41 |  |  | 0.156 | 0.00359 | 480293.33 | 11046.75 |
| Ozone formation, terrestrial ecosystems | -3.07E-13 | 7.06E-15 | 2278.5 | 52.41 |  |  | 0.156 | 0.00359 | 480293.33 | 11046.75 |
| Terrestrial acidification | -1.10E-13 | 2.54E-15 | 820.26 | 18.87 |  |  | 0.0562 | 0.00129 | 172905.6 | 3976.83 |
| Terrestrial ecotoxicity |  |  |  |  |  |  | 6.59E-05 | 1.52E-06 |  |  |

Table S21. Base results and standard deviation of different impact categories contributed by the respective processes in Singapore derived using ReCiPe midpoint (H) method. The largest score value of data quality indicator of 4 were selected for all quality aspects to derive for the standard deviations (C.V.= 103.5 %).

| **Processes (Standard deviation @ DQI =4)** | **Bundled PET Bottles to Waste** | | **Used bottle to Traders/MRF** | | **Mechanical recycling** | | **Incineration** | | **Landfill** | |
| --- | --- | --- | --- | --- | --- | --- | --- | --- | --- | --- |
|  | **Base result** | **Standard deviation** | **Base result** | **Standard deviation** | **Base result** | **Standard deviation** | **Base result** | **Standard deviation** | **Base result** | **Standard deviation** |
| Fine particulate matter formation | -3.37E-14 | -3.49E-14 | 250.64 | 259.31 |  |  | 0.0172 | 0.0178 | 52832.27 | 54660.46 |
| Fossil resource scarcity |  |  |  |  | 1151957520 | 1191819615 | 1.59E-07 | 1.65E-07 |  |  |
| Freshwater ecotoxicity |  |  |  |  |  |  | 3.50E-08 | 3.62E-08 |  |  |
| Global warming |  |  |  |  | 12152000000 | 12572505243 | 19.04 | 19.70 | 6427250667 | 6649657892 |
| Human carcinogenic toxicity |  |  |  |  |  |  | 2.55 | 2.64 |  |  |
| Human non-carcinogenic toxicity |  |  |  |  |  |  | 0.954 | 0.987 |  |  |
| Marine ecotoxicity |  |  |  |  |  |  | 6.83E-07 | 7.06E-07 |  |  |
| Ozone formation, human health | -3.07E-13 | -3.17E-13 | 2278.5 | 2357.35 |  |  | 0.156 | 0.161 | 480293.33 | 496913.30 |
| Ozone formation, terrestrial ecosystems | -3.07E-13 | -3.18E-13 | 2278.5 | 2357.35 |  |  | 0.156 | 0.161 | 480293.33 | 496913.30 |
| Terrestrial acidification | -1.10E-13 | -1.14E-13 | 820.26 | 848.64 |  |  | 0.0561 | 0.0581 | 172905.6 | 178888.79 |
| Terrestrial ecotoxicity |  |  |  |  |  |  | 6.59E-05 | 6.81E-05 |  |  |

Table S22. Base results and standard deviation of different impact categories contributed by the respective processes in India derived using ReCiPe midpoint (H) method. The smallest score value of data quality indicator of 1 were selected for all quality aspects to derive for the standard deviations (C.V.= 2.3 %).

| **Processes (Standard deviation @ DQI =1)** | **Bundled PET Bottles to Waste** | | **Used bottle to Traders/MRF** | | **Mechanical recycling** | | **Incineration** | | **Landfill** | | **Cement kiln co-processing** | |
| --- | --- | --- | --- | --- | --- | --- | --- | --- | --- | --- | --- | --- |
|  | **Base result** | **Standard deviation** | **Base result** | **Standard deviation** | **Base result** | **Standard deviation** | **Base result** | **Standard deviation** | **Base result** | **Standard deviation** | **Base result** | **Standard deviation** |
| Fine particulate matter formation | 3.23E-11 | 7.44E-13 | 132.89 | 3.06 | -7.42E-12 | 1.71E-13 |  |  |  |  | -884861.63 | 20351.82 |
| Freshwater ecotoxicity |  |  | -3278.43 | 75.40 |  |  |  |  |  |  | -3278.43 | 75.40 |
| Global warming | 3.42E-10 | 7.87E-12 |  | 0 | -8.79E-08 | 2.02E-09 | -2.03E-05 | 4.68E-07 | 4.89E+12 | 1.13E+11 |  |  |
| Human carcinogenic toxicity |  |  |  |  |  |  |  |  |  |  | -225919.28 | 5196.14 |
| Human non-carcinogenic toxicity |  |  |  |  |  |  |  |  |  |  | -80499971.42 | 1851499.34 |
| Marine ecotoxicity |  |  |  |  |  |  |  |  |  |  | -779031.98 | 17917.74 |
| Ozone formation, human health | 2.94E-10 | 6.76E-12 | 1209 | 27.79 | -5.56E-11 | 1.28E-12 | -4.66E-08 | 1.07E-09 |  |  | -4585623.84 | 105469.35 |
| Ozone formation, terrestrial ecosystems | 2.94E-10 | 6.76E-12 | 1208.10 | 27.79 | -5.56E-11 | 1.28E-12 | -7.52E-08 | 1.73E-09 |  |  | -4585623.84 | 105469.35 |
| Terrestrial acidification | 1.06E-10 | 2.43E-12 | 434.92 | 10 | -2.46E-11 | 5.65E-13 |  |  |  |  | -2974104.61 | 68404.41 |

Table S23. Base results and standard deviation of different impact categories contributed by the respective processes in India derived using ReCiPe midpoint (H) method. The largest score value of data quality indicator of 4 were selected for all quality aspects to derive for the standard deviations (C.V.= 103.5 %).

| **Processes (Standard deviation @ DQI = 4)** | **Bundled PET Bottles to Waste** | | **Used bottle to Traders/MRF** | | **Mechanical recycling** | | **Incineration** | | **Landfill** |  | **Cement kiln co-processing** | |
| --- | --- | --- | --- | --- | --- | --- | --- | --- | --- | --- | --- | --- |
|  | **Base result** | **Standard deviation** | **Base result** | **Standard deviation** | **Base result** | **Standard deviation** | **Base result** | **Standard deviation** | **Base result** | **Standard deviation** | **Base result** | **Standard deviation** |
| Fine particulate matter formation | 3.23E-11 | 3.34E-11 | 132.89 | 137.49 | -7.42E-12 | 7.68E-12 |  |  |  |  | -884861.69 | 915481.19 |
| Freshwater ecotoxicity |  |  | -3278.43 | 3391.87 |  |  |  |  |  |  | -3278.43 | 3391.87 |
| Gloal warming | 3.42E-10 | 3.54E-10 |  |  | -8.79E-08 | 9.10E-08 | -2.03E-05 | 2.11E-05 | 4.89E+12 | 5.06E+12 |  |  |
| Human carcinogenic toxicity |  |  |  |  |  |  |  |  |  |  | -225919.28 | 233736.94 |
| Human non-carcinogenic toxicity |  |  |  |  |  |  |  |  |  |  | -80499971.42 | 83285575.44 |
| Marine ecotoxicity |  |  |  |  |  |  |  |  |  |  | -779031.98 | 805989.44 |
| Ozone formation, human health | 2.94E-10 | 3.04E-10 | 1208.10 | 1249.91 | -5.56E-11 | 5.76E-11 | -4.66E-08 | 4.83E-08 |  |  | -4585623.84 | 4744303.80 |
| Ozone formation, terrestial ecosystems | 2.94E-10 | 3.04E-10 | 1208.10 | 1249.91 | -5.56E-11 | 5.76E-11 | -7.52E-08 | 7.78E-08 |  |  | -4585623.84 | 4744303.80 |
| Terrestial acidification | 1.06E-10 | 1.1E-10 | 434.92 | 449.97 | -2.46E-11 | 2.54E-11 |  |  |  |  | -2974104.61 | 3077019.89 |

Table S24. Base results and standard deviation of different impact categories contributed by the respective processes for manufacturing TENG in laboratory- and industrial-scale setup derived using ReCiPe midpoint (H) method. The smallest score value of data quality indicator of 1 were selected for all quality aspects to derive for the standard deviations (C.V.= 2.3 %), and the largest score value of data quality indicator of 4 were selected for all quality aspects to derive for the standard deviations (C.V.= 103.5 %).

| **Manufacturing processes** | **Stirring** | | | **Electrospinning** | | | **Autoclaving** | | | **Centrifugation** | | | **Freeze drying** | | |
| --- | --- | --- | --- | --- | --- | --- | --- | --- | --- | --- | --- | --- | --- | --- | --- |
|  | **Base result** | **Standard deviation** | | **Base result** | **Standard deviation** | | **Base result** | **Standard deviation** | | **Base result** | **Standard deviation** | | **Base result** | **Standard deviation** | |
|  |  | **DQI = 1**  **(CV = 2.3%)** | **DQI = 4**  **(CV = 103.4%)** |  | **DQI = 1**  **(CV = 2.3%)** | **DQI = 4**  **(CV = 103.4%)** |  | **DQI = 1**  **(CV = 2.3%)** | **DQI = 4**  **(CV = 103.4%)** |  | **DQI = 1**  **(CV = 2.3%)** | **DQI = 4**  **(CV = 103.4%)** |  | **DQI = 1**  **(CV = 2.3%)** | **DQI = 4**  **(CV = 103.4%)** |
| **Laboratory-scale manufacturing** | | | | | | | | | | | | | | | |
| Fossil resource scarcity  (kg oil eq) | -522.72 | 12.02 | 540.49 | -475.2 | 10.93 | 491.36 | -35.64 | 0.82 | 36.85 | -2.178 | 0.05 | 2.25 | -4181.76 | 96.18 | 4323.94 |
| Global warming  (kg CO2 eq) | -313.632 | 7.21 | 324.30 | -285.12 | 6.56 | 294.81 | -21.384 | 0.49 | 22.11 | -1.3068 | 0.03 | 1.35 | -2509.06 | 57.71 | 2594.37 |
| **Industrial-scale manufacturing** | | | | | | | | | | | | | | | |
| Fossil resource scarcity  (kg oil eq) | -1,980 | 45.54 | 2,047.32 | -2,639,894 | 60,717.56 | 2,729,650.40 | -15,286 | 351.58 | 15,805.72 | -712.8 | 16.39 | 737.04 | -3,801,600 | 87,436.8 | 3,930,854.4 |
| Global warming  (kg CO2 eq) | -11.88 | 0.237 | 12.28 | -15,832.7 | 364.15 | 16,371.01 | -91.68 | 2.11 | 94.80 | -4.28 | 0.098 | 4.43 | -22,800 | 52.44 | 23,575.2 |

***Reference table***

| **Process** | **Literature study pertaining to performance, consumption, and emission** | **Reference** |
| --- | --- | --- |
| Centrifugation | ANDRITZ Pusher Centrifuge (SZ 800/2)  Capacity: 14,000 kg/h absolute dry solid throughput. Input solid concentration-15wt%  Electricity consumption 9kW/t (40kW per hour) | <https://www.process-worldwide.com/filtration-equipment-selection-criteria-a-300953/?p=3> |
| Autoclave | AMSCO Evolution Steam Steriliser (HC1500)  (Consumption for 1 ton = 27.23kW)  Size: 660mm x 950mm x 1676mm  Autoclaving/sterilizer Capacity:  45 kg per cycle  141 kg per hr  Water consumption (considering water re-circulating):  473 litres per cycle  2.08 litres per kg  (2080 litres per ton) | <https://www.ncbi.nlm.nih.gov/pmc/articles/PMC7178787/>  <https://cleanroomtechnology.com/news/article_page/Autoclave_packaging_for_efficient_sterilisation/155556>  <https://www.steris.com/healthcare/knowledge-center/sterile-processing/everything-about-autoclaves>  <https://www.steris.com/healthcare/products/steam-sterilizers/amsco-evolution-steam-sterilizer> |
| Electrospinning | Elmarco’s Nanospider Production Line (NS 8S1600U)  Electrical consumption: max. 5 kW (83.33 kW per ton)  Volume of solution per batch: 60 Litres  Example: 20 000 000 m2/year for PA6 on cellulose,  nanofiber layer width: 1.6 m,  basis weight: 0,03 g/m2, fiber diameter: 150 nm +/- 30%,  85% uptime | https://doi.org/10.1002/mame.201200290  https://www.elmarco.com/production-lines/ns-8s1600u |
| Stirring | IKA Stirring (Standard Production Plant, SPP 1000)  Maximum: 1000 litres  Electrical consumption: 25 kW | <https://www.azom.com/article.aspx?ArticleID=14131>  https://www.ikaprocess.com/en/Products/Process-plants-cph-18/Standard-Plant-SPP-csb-SPP/Technical-Data-cspt.html |
| Freeze drying | Cuddon Freeze Dryer FD1500  Capacity: 1500kg ice capacity in 24hr period, 137m2 area  Electricity consumption: 2kWh/kg of wet product  (2000 kW per ton)  Size:  7.15m long x 2.79m wide x 2.47m high  Weight(unloaded) 20,400kg | http://www.cuddonfreezedry.com/assets/uploads/2018/05/FD1500-Gen-Spec-2018.pdf |
